# Biofilm-dispersed pneumococci induce elevated leukocyte and platelet activation

**DOI:** 10.3389/fcimb.2024.1405333

**Published:** 2024-08-01

**Authors:** Yashuan Chao, Martina Mørch, Anders P. Håkansson, Oonagh Shannon

**Affiliations:** ^1^ Division of Infection Medicine, Department of Clinical Sciences, Lund, Faculty of Medicine, Lund University, Lund, Sweden; ^2^ Section for Oral Biology, Faculty of Odontology, Malmö University, Malmö, Sweden; ^3^ Division of Experimental Infection Medicine, Department of Translational Medicine, Faculty of Medicine, Lund University, Lund, Sweden

**Keywords:** leukocyte activation, monocytes, neutrophils, platelets, platelet activation, *Streptococcus pneumoniae*

## Abstract

**Introduction:**

*Streptococcus pneumoniae* (the pneumococcus) effectively colonizes the human nasopharynx, but can migrate to other host sites, causing infections such as pneumonia and sepsis. Previous studies indicate that pneumococci grown as biofilms have phenotypes of bacteria associated with colonization whereas bacteria released from biofilms in response to changes in the local environment (i.e., dispersed bacteria) represent populations with phenotypes associated with disease. How these niche-adapted populations interact with immune cells upon reaching the vascular compartment has not previously been studied. Here, we investigated neutrophil, monocyte, and platelet activation using *ex vivo* stimulation of whole blood and platelet-rich plasma with pneumococcal populations representing distinct stages of the infectious process (biofilm bacteria and dispersed bacteria) as well as conventional broth-grown culture (planktonic bacteria).

**Methods:**

Flow cytometry and ELISA were used to assess surface and soluble activation markers for neutrophil and monocyte activation, platelet-neutrophil complex and platelet-monocyte complex formation, and platelet activation and responsiveness.

**Results:**

Overall, we found that biofilm-derived bacteria (biofilm bacteria and dispersed bacteria) induced significant activation of neutrophils, monocytes, and platelets. In contrast, little to no activation was induced by planktonic bacteria. Platelets remained functional after stimulation with bacterial populations and the degree of responsiveness was inversely related to initial activation. Bacterial association with immune cells followed a similar pattern as activation.

**Discussion:**

Differences in activation of and association with immune cells by biofilm-derived populations could be an important consideration for other pathogens that have a biofilm state. Gaining insight into how these bacterial populations interact with the host immune response may reveal immunomodulatory targets to interfere with disease development.

## 1 Introduction

*Streptococcus pneumoniae*, or the pneumococcus, is an effective colonizer of the human nasopharynx. Asymptomatic colonization of the nasopharynx is the most common outcome of acquisition and is thought to precede pneumococcal dissemination and disease (Bogaert et al., 2004; Short and Diavatopoulos, 2015). Pneumococcal dissemination is highly associated with disease triggers such as upper respiratory virus infections or other assaults that activate local inflammation (Short et al., 2012; Bosch et al., 2013; Teo et al., 2015; Bakaletz, 2017; Jusot et al., 2017). In the presence of disease triggers, bacteria can migrate to and survive and cause infection in otherwise non-infected host sites, such as the middle ears and lungs, resulting in acute otitis media and pneumonia. Pneumococci that reach the bloodstream can cause sepsis—a life-threatening condition resulting from a dysregulated immune response to infection. The transition from colonization to disease occurs frequently enough that the pneumococcus remains a major cause of death worldwide, especially in susceptible populations such as children and elderly individuals (O’Brien et al., 2009).

Much like other respiratory pathogens, pneumococci form biofilms during colonization of the upper respiratory tract (Hall-Stoodley et al., 2006; Munoz-Elias et al., 2008; Hoa et al., 2009; Marks et al., 2012; Blanchette-Cain et al., 2013). Bacterial biofilms are communities of bacteria encased within a self-produced extracellular matrix composed of polysaccharides, proteins, lipids, and extracellular DNA (Flemming et al., 2016). Disease triggers, such as viral infection or virus-induced host responses (including fever), can induce the release of pneumococci from biofilms (Marks et al., 2013). These dispersed bacteria have a distinct transcriptional profile displaying the gene expression of proteins associated with protection from host inflammation and antagonistic surrounding microflora (Marks et al., 2013; Pettigrew et al., 2014). Dispersed bacteria, as compared with biofilm bacteria or conventional broth-grown planktonic bacteria, represent a distinct population that exhibit less adherence, more invasion, and more toxicity to human respiratory epithelial cells *in vitro*, eliciting higher levels of proinflammatory cytokines from exposed epithelial cells (Marks et al., 2013). In a murine pneumonia model, challenge with dispersed bacteria leads to a higher bacterial burden and more leukocyte infiltrate in the lungs as well as more dissemination to the bloodstream than both biofilm and planktonic populations (Marks et al., 2013). Moreover, dispersed bacteria are hypervirulent in a murine sepsis model, suggesting that the dispersed phenotype may play a role in the survival of pneumococci during the development of acute infection in the vascular compartment (Marks et al., 2013). Altogether, these studies have been fundamental in our understanding of signal-induced pneumococcal biofilm dispersion and the changed phenotype in dispersed bacteria, providing important insight into the transition from pneumococcal colonization to disease.

Upon entry into the bloodstream, pneumococci encounter innate immune cells, such as neutrophils, monocytes, and platelets, that play an important role in the host immune defense against bacterial pathogens. Activated leukocytes upregulate CD11b expression on the cell surface and can also release myeloperoxidase (MPO; mostly from azurophilic granules of neutrophils). Platelets are well known for their critical role in hemostasis, but like neutrophils and monocytes, platelets can recognize “danger signals” via pattern recognition receptors and have important roles in infection and inflammation (Kapur et al., 2015). Indeed, platelets have been reported to have a protective role in mouse models of pneumococcal infection, as platelet depletion leads to enhanced bacterial growth and increased mortality (van den Boogaard et al., 2015; Schrottmaier et al., 2022). Activated platelets can interact both directly with pathogens or immune cells, such as leukocytes, or indirectly via the production of inflammatory mediators to regulate pathogen survival or the host immune response (Rossaint and Zarbock, 2015). Direct interaction through platelet-neutrophil complex (PNC) and platelet-monocyte complex (PMC) formation is often used as a marker for platelet activation in blood. Furthermore, platelet-free and platelet-bound leukocytes can differ in activation status upon stimulation with bacteria or bacterial products (Nicu et al., 2009; Stahl et al., 2009).

Platelets have various receptor-ligand specific signaling pathways that, ultimately, converge to stimulate platelet shape change and platelet granule secretion (Li et al., 2010). Platelets contain storage granules and exocytosis of these granules is central to platelet function. α-granules are the most abundant secretory organelles in platelets, followed by dense granules. α-granules and dense granules contain membrane proteins, such as CD62P and CD63, and soluble cargo, such as platelet factor 4 (PF4) and adenosine diphosphate (ADP), respectively (Heijnen and van der Sluijs, 2015; Sharda and Flaumenhaft, 2018). Upon platelet activation, granule exocytosis results in the release of cargo and granule membrane proteins are translocated to the platelet surface membrane. Surface CD62P can be shed from the platelet surface, which correlates with increased levels of soluble CD62P in plasma (Michelson et al., 1996; Berger et al., 1998).

Although biofilm bacteria and biofilm-dispersed bacteria represent populations potentially more closely resembling the phenotypes of bacteria during colonization and transition to disease, broth-grown planktonic pneumococci are commonly used in experimental studies. Studies on platelet activation by bacteria, including pneumococci (Keane et al., 2010; de Stoppelaar et al., 2016; Wolff et al., 2020), have been conducted with planktonic bacteria grown in nutrient-rich media at 37°C. Platelet activation in response to pneumococci specifically adapted to various host niches has not been studied.

Here, we focus on the acute inflammatory response to pneumococcal populations associated with colonization (biofilm bacteria), transition to disease (dispersed bacteria), and conventional culture conditions (planktonic bacteria) to evaluate potential differences associated with bacteria adapted to various niches. Based on the phenotypes of these populations in previous murine infection models (Marks et al., 2013), we hypothesize that the inflammatory responses will be distinct, with biofilm bacteria causing low levels of inflammation, dispersed bacteria causing hyperinflammation, and planktonic bacteria causing moderate inflammation. We investigate neutrophil, monocyte, and platelet activation using *ex vivo* stimulation of whole blood and platelet-rich plasma (PRP) with pneumococcal populations of the clinical isolate EF10175 or the well-studied laboratory strain D39. We investigate the activation of neutrophils and monocytes and the formation of PNCs and PMCs in whole blood, activation of platelets in PRP, and association of bacteria with immune cells. Additionally, we assess the impact of activated platelets on neutrophil activation status, platelet functionality, and bacterial association to neutrophils.

## 2 Materials and methods

### 2.1 Reagents

Cell culture reagents were purchased from GE Healthcare Life Sciences. Phosphate-buffered saline (PBS; 0.14 M NaCl, 0.0027 M KCl, 0.0101 M phosphate buffer, pH 7.4; Medicago) was purchased from Saveen & Werner. Paraformaldehyde, HEPES, NaCl, adenosine diphosphate (ADP), N-formylmethionine-leucyl-phenylalanine (fMLF), thrombin receptor activator peptide 6 (TRAP-6), quinacrine dihydrochloride (mepacrine), prostaglandin E1, and Triton X-100 were purchased from Sigma. KCl, MgSO_4_, and formaldehyde (37%) were purchased from Merck. Anticoagulant peptide H-Gly-Pro-Arg-Pro-NH_2_ acetate salt (Cat. no. H-1998) was purchased from Bachem. Thrombin was purchased from Chrono-log. Oregon Green 488-X, succinimidyl ester, 6-isomer (OG488-X) and eBioscience 1-step Fix/Lyse Solution (10X) were purchased from Invitrogen. The following fluorescently conjugated antibodies were purchased from BD Pharmingen: CD11b-PE-Cy™5 (clone ICRF44), CD61-PE (clone VI-PL2), CD63-Alexa Fluor^®^ 647 (clone H5C6); BD Biosciences: CD42a-PerCP (clone Beb1) and CD62P-PE (clone AC1.2); and eBioscience, Invitrogen, ThermoFisher Scientific: CD45-APC (cloneHI30) and CD45-FITC (clone HI30).

### 2.2 Preparation of bacterial populations

The following pneumococcal strains were used in this study: clinical isolate EF10175 (Andersson et al., 1983) and laboratory strain D39 (Avery et al., 1944). Biofilm, biofilm-dispersed (triggered by febrile temperature), and broth-grown planktonic pneumococcal populations were prepared as previously described (Chao et al., 2019, Chao et al., 2020). Among the tested host signals associated with viral infection, a febrile temperature of 38.5°C (an elevated temperature compared to the biofilm growth temperature of 34°C) alone led to the most biofilm dispersal and caused a phenotypic change in dispersed bacteria that was representative for the responses observed by other stimuli (Marks et al., 2013; Pettigrew et al., 2014). Pneumococcal populations were stored in 13% glycerol at -80°C. Biofilm formation and biofilm dispersal were confirmed by viable plate counts as previously described (Chao et al., 2020). Biofilm biomass, gentamicin sensitivity, and dispersal were comparable to previously described values (**Supplementary Table 1**) (Chao et al., 2020).

Prior to assays, frozen glycerol stocks of bacterial populations were thawed on ice, washed twice in cold HEPES buffer (referred to as HEPES; pH 7.4, 10 mM HEPES, 150 mM NaCl, 5 mM KCl, 1 mM MgSO_4_), and resuspended in cold HEPES to a final concentration of 1 x 10^8^ CFU/ml. In some experiments, bacteria were labeled with OG488-X: Thawed glycerol stocks were incubated with 1/10 volume of either HEPES or 200 µM OG488-X for 30 min on ice prior to the wash and resuspension steps. OG488-X staining did not affect bacterial viability (**Supplementary Table 2**).

### 2.3 Whole blood and platelet activation assays

The recruitment of healthy donors was approved by The Regional Ethical Review Authority, Lund (approval number 2015/801). Whole blood was obtained in BD Vacutainer^®^ citrate tubes (Cat. no. 357714). Platelet-rich plasma (PRP) was prepared from citrated whole blood by centrifugation at 150 x *g* for 10 min and was used in assays within 4 h of blood collection.

Citrated whole blood (45 µl) was stimulated with a 5 µl volume of 1 x 10^8^ CFU/ml of bacteria (final concentration of 1 x 10^7^ CFU/ml), physiological agonists (final concentrations of 1 U/ml thrombin or 1 µM fMLF), or HEPES buffer (to determine baseline level) for 15 min at 37°C. Prior to the addition of thrombin, the synthetic anticoagulant peptide H-Gly-Pro-Arg-Pro-NH_2_ was added to a final concentration of 2.5 mg/ml to prevent fibrin clot formation. Subsequent steps were performed at room temperature and in the dark. Samples were stained (1/10) with CD45-FITC (or CD45-APC in experiments with stained bacteria; gating neutrophils and monocytes), CD61-PE (platelet-positive events), and CD11b-PE-Cy™5 (leukocyte activation) for 15 min, fixed using 1 ml of 1X 1-step Fix/Lyse Solution for 1 h, and centrifuged at 500 x *g* for 5 min. One ml of supernatant was carefully removed and discarded, and the remaining volume with cells was resuspended in 500 µl of HEPES and analyzed by flow cytometry. Stimulation with bacterial populations did not affect the total number of neutrophils or monocytes per sample as assessed by events/µl of sample (**Supplementary Figures 1A, B**).

PRP was diluted 1/10 in HEPES buffer. 20 µl of 1/10 diluted PRP was added to 25 µl of HEPES (referred to as diluted PRP) and stimulated with a 5 µl volume of 1 x 10^8^ CFU/ml of bacteria (final concentration of 1 x 10^7^ CFU/ml), physiological agonists (final concentrations of 5 µM ADP or 2.5 mg/ml H-Gly-Pro-Arg-Pro-NH_2_ + 1 U/ml thrombin), or HEPES buffer for 15 min at 37°C. In some experiments, stimulated PRP was incubated for an additional 5 min at 37°C in the presence of 20 μM TRAP-6 or vehicle control. Samples were stained with CD42a-PerCP (1/10; resting and activated platelets), CD62P-PE (1/10; platelet activation), and CD63-Alexa Fluor^®^ 647 (1/50; platelet activation) for 15 min. In some experiments, samples were incubated with antibodies in the presence of 1 µM mepacrine (green fluorescent dye) or vehicle control (see below). Samples were fixed with 500 µl of 1% (v/v in HEPES from 37% stock) formaldehyde for 1 h and analyzed by flow cytometry. Stimulation with bacterial populations did not affect the total number of platelets per sample as assessed by events/µl of sample (**Supplementary Figure 1C**). In an initial screening, stimulation of platelets in PRP for up to 60 min was tested, but the activation level was the same as obtained at 15 min (data not shown). For logistical reasons, shorter timepoints were not tested. For consistency between assays, the same experimental parameters (15 min at 37°C) were used for whole blood stimulation assays.

To generate immune cell releasates for ELISA analysis of granule release, a volume of 250 µl of citrated whole blood or diluted PRP (see above) was stimulated with control agonists or pneumococcal populations in proportional volumes as described above for 15 min at 37°C. To prevent the activation of platelets during centrifugation, 3.5 µl of 10 µg/ml prostaglandin E1 was added to stimulated samples and incubated for 2 min at room temperature. Plasma was prepared by centrifugation at 2000 x *g* for 10 min and stored at -20°C until use.

### 2.4 Bacterial viability assay

Diluted PRP was stimulated with H-Gly-Pro-Arg-Pro-NH_2_ and thrombin (see above) for 5 min at 37°C to obtain thrombin-activated PRP (PRP-Thr). Diluted PRP and PRP-Thr were centrifuged at 2000 x *g* for 10 min to obtain platelet-poor plasma (PPP) and thrombin-activated PPP (PPP-Thr), respectively. For bacterial viability analysis, a volume of 72 µl of HEPES (untreated), PRP, PRP-Thr, PPP, or PPP-Thr was incubated with 8 µl of 1 x 10^8^ CFU/ml of bacteria (final concentration of 1 x 10^7^ CFU/ml) for 15 min at 37°C. Bacterial viability was determined by viable counts on blood agar plates. Platelets and platelet releasates (either unstimulated or thrombin-activated) did not kill the pneumococcal populations in the tested conditions (**Supplementary Figure 2**).

### 2.5 Flow cytometry

Fixed samples were analyzed on a BD Accuri C6 Plus flow cytometer and software (BD Biosciences). For whole blood, linear settings were applied, the FSC-H threshold was set at 150,000, and 20,000 events were collected per sample excluding red blood cell debris. For PRP, logarithmic settings were applied, the FSC-H threshold was set at 45,000, and 100,000 events were collected per sample in the CD42a-positive platelet gate. Representative gating strategies and histograms are presented in **Supplementary Figure 3** for whole blood and in **Supplementary Figure 4** for PRP. Gating strategies used here for neutrophils and monocytes in whole blood and platelets in PRP are commonly used and have previously been published to define the cells of interest in our experimental setup (Chao et al., 2021; Palm et al., 2022). Data are shown as median fluorescence intensity (MFI) relative to the baseline control (HEPES) unless otherwise stated. Innate immune cells are highly reactive, therefore, using a relative MFI (where values are normalized to baseline) accounts for baseline variation among individuals and experiments.

Mepacrine release and uptake: To investigate platelet dense granule release and residual dense granule content after stimulation with agonists or bacteria, mepacrine was used as a green fluorescent marker in PRP experiments (**Supplementary Figure 4C**). Mepacrine binds to adenosine nucleotides and is rapidly taken up by platelet dense granules (Irvin and Irvin, 1954; Wall et al., 1995). Using platelet samples incubated with mepacrine, mepacrine release (i.e., platelet dense granule release) was calculated by subtracting the MFI of the sample stimulated with HEPES (baseline) from the MFI of each stimulated sample. Mepacrine uptake (i.e., residual dense granule content) was calculated for each agonist or bacterial population as the difference of MFI between platelet samples incubated with mepacrine and platelet samples incubated with vehicle (background), and the data is shown relative to the baseline control (HEPES).

Bacterial association with immune cells was determined in experiments with bacteria pre-labeled with OG488-X (green fluorescent conjugate). The association of bacteria with immune cells was assessed by measuring the percentage of FITC-positive neutrophils, monocytes, or platelets (**Supplementary Figures 3D, E**, **4D**). Samples with non-stained bacteria were used as negative controls. The percentage of bacteria association was determined by calculating the difference between the percentages of samples stimulated with OG488-X-stained bacteria and non-stained bacteria.

### 2.6 ELISA

ELISA kits (R&D Systems) were used to measure plasma levels of MPO (1/200 dilution; DY3174), soluble CD62P (1/20 dilution; DY137), and PF4 (1/5000 dilution; DY795) according to manufacturer’s instructions, except 100 µl of ELISA Stop Solution (Invitrogen) was used to stop the reaction. MPO was measured in plasma prepared from whole blood and soluble CD62P and PF4 were measured in plasma prepared from PRP.

### 2.7 Statistics

Statistical analyses were performed using GraphPad Prism 10 software. Comparisons were analyzed for statistical significance using Wilcoxon matched-pairs signed rank test or Friedman test with Dunn’s multiple comparisons test. Results were deemed significant for comparisons where *P* < 0.05.

## 3 Results

**Figure 1:**
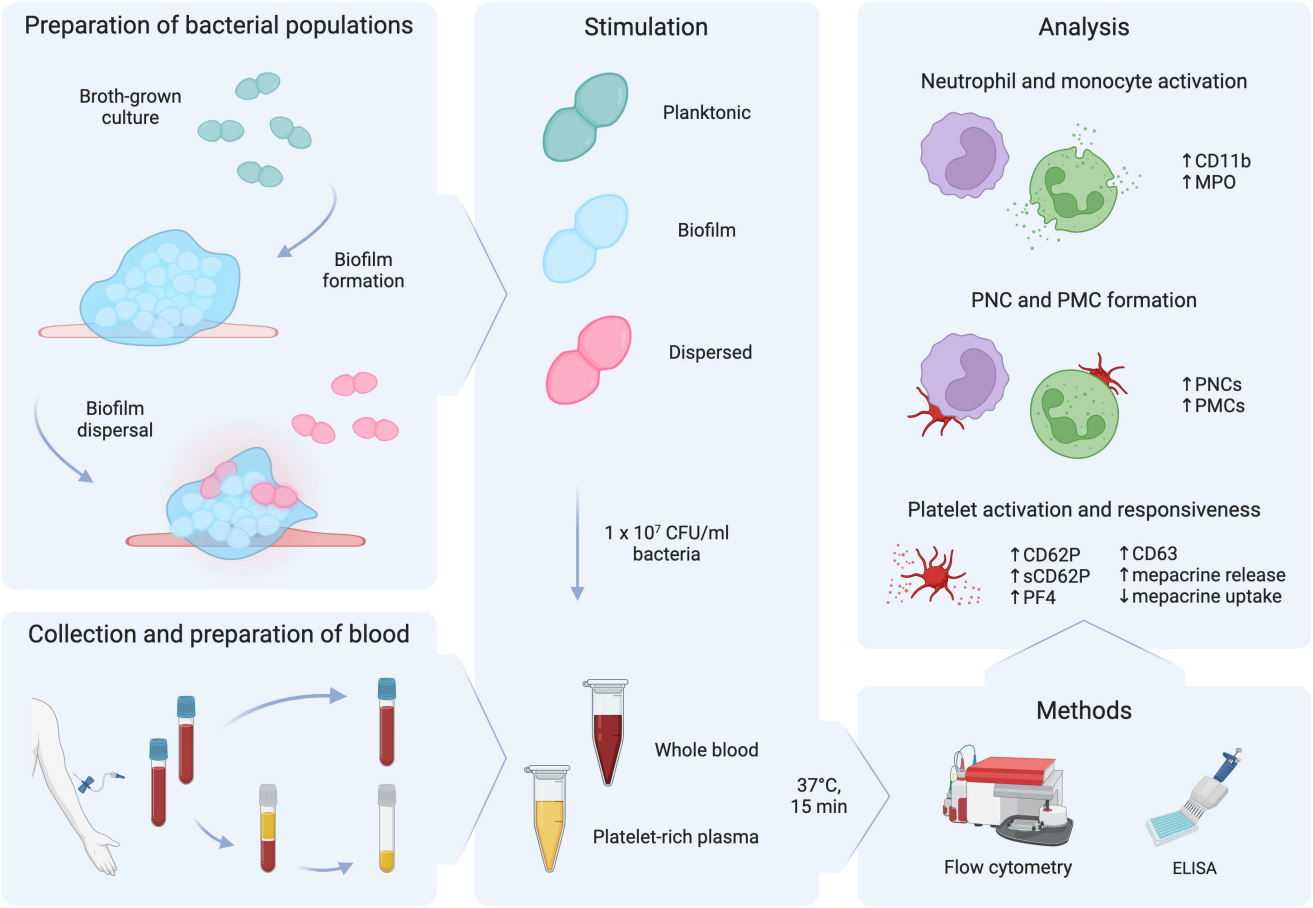
Schematic diagram of the workflow. Planktonic bacteria grown in broth cultures were used to seed biofilms. After biofilm formation, biofilms were exposed to a febrile temperature of 38.5°C to trigger biofilm dispersal. Citrated whole blood was obtained from healthy donors and was used to prepare platelet-rich plasma. Whole blood and platelet-rich plasma were stimulated with 1 x 10^7^ CFU/ml of planktonic, biofilm, or dispersed populations of pneumococci for 15 min at 37°C. Flow cytometry and ELISA were used to measure regulation of markers for neutrophil and monocyte activation, platelet-neutrophil complex (PNC) and platelet-monocyte (PMC) formation, and platelet activation and responsiveness. This figure was created with BioRender.com.

Based on previous findings demonstrating signal-induced pneumococcal dispersion and subsequent phenotypic changes in dispersed bacteria, we focused on investigating the innate immune response by neutrophils, monocytes, and platelets to these niche-distinct pneumococcal populations. Whole blood and platelet-rich plasma were stimulated *ex vivo* with biofilm bacteria (associated with colonization), dispersed bacteria (associated with transition to disease), and planktonic bacteria (associated with conventional broth-grown culture conditions) as illustrated in **Figure 1**.

### 3.1 Neutrophil and monocyte activation in blood

**Figure 2:**
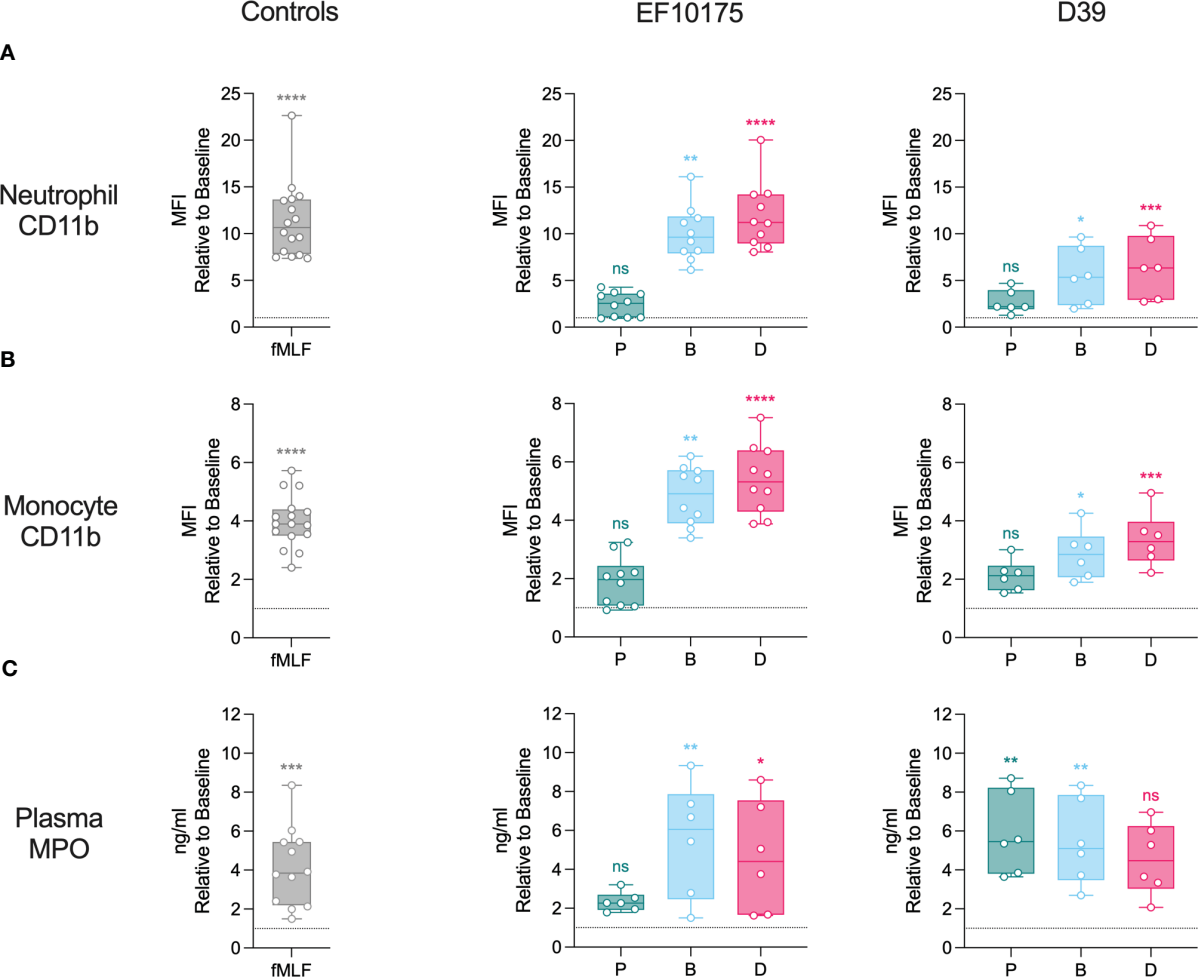
Neutrophil and monocyte activation in whole blood. Citrated whole blood was stimulated with HEPES (to determine baseline levels), 1 μM fMLF, or populations of planktonic (P), biofilm (B), or dispersed (D) pneumococci of strain EF10175 or strain D39 (1 x 10^7^ CFU/ml) for 15 min at 37°C. The median fluorescent intensity (MFI) of activation marker CD11b on **(A)** neutrophils and **(B)** monocytes were determined by flow cytometry. **(C)** Plasma levels of MPO were determined by ELISA. Flow cytometry: EF10175, n = 10 independent experiments from 8 healthy donors; D39, n = 6 independent experiments from 5 healthy donors. ELISA: EF10175, n = 6 independent experiments from 6 healthy donors; D39, n = 6 independent experiments from 5 healthy donors. Data are shown relative to baseline with the median ± interquartile range displayed. Statistical analysis was performed using Wilcoxon matched-pairs signed rank test or Friedman test with Dunn’s multiple comparisons test; ns, not significant; *P < 0.05, **P < 0.01, ***P < 0.001, ****P < 0.0001.

To investigate the activation of classical innate immune cells of the blood compartment, whole blood was stimulated *ex vivo* with leukocyte agonist fMLF or pneumococcal populations of strains EF10175 and D39. Flow cytometry was used to determine upregulation of CD11b on the surface of neutrophils (**Figure 2A**) and monocytes (**Figure 2B**). As expected, fMLF induced a significant increase of CD11b on neutrophils and monocytes relative to the baseline control (HEPES). Both dispersed bacteria and biofilm bacteria induced significant increases in CD11b on both cell types, whereas planktonic populations of both strains induced a low level of activation that was not significantly different from baseline. Levels of CD11b induced by dispersed bacteria and biofilm bacteria of strain EF10175 were similar to that observed with the positive control fMLF, and were higher than the response to respective populations of strain D39. The fold increase in CD11b by dispersed bacteria was significantly higher than by biofilm bacteria for both strain EF10175 and strain D39 (**Supplementary Table 3**).

Activated leukocytes (primarily neutrophils) release myeloperoxidase (MPO). As an additional measure of leukocyte activation, plasma levels of MPO were determined using ELISA (**Figure 2C**; **Supplementary Table 3**). MPO was significantly increased following stimulation with fMLF. Dispersed bacteria and biofilm bacteria of strain EF10175 induced significantly higher levels of MPO compared to baseline, while planktonic bacteria induced low levels. In contrast, all three populations of strain D39 induced elevated MPO levels.

These results demonstrate activation of blood leukocytes by bacterial populations associated with distinct stages of the infectious process (biofilm bacteria and dispersed bacteria) that contrasts with the lack of significant activation by conventional broth-grown bacteria (planktonic bacteria).

### 3.2 Platelet-leukocyte complex formation in blood

Activated platelets can interact with leukocytes to form platelet-leukocyte complexes, a surrogate marker for platelet activation in blood. To investigate platelet-leukocyte formation, whole blood was stimulated *ex vivo* with platelet agonist thrombin, leukocyte agonist fMLF, or pneumococcal populations of strains EF10175 and D39. Flow cytometry was used to determine platelet-neutrophil complex (PNC) and platelet-monocyte complex (PMC) formation by measuring platelet CD61 staining on neutrophils and monocytes, respectively.

**Figure 3:**
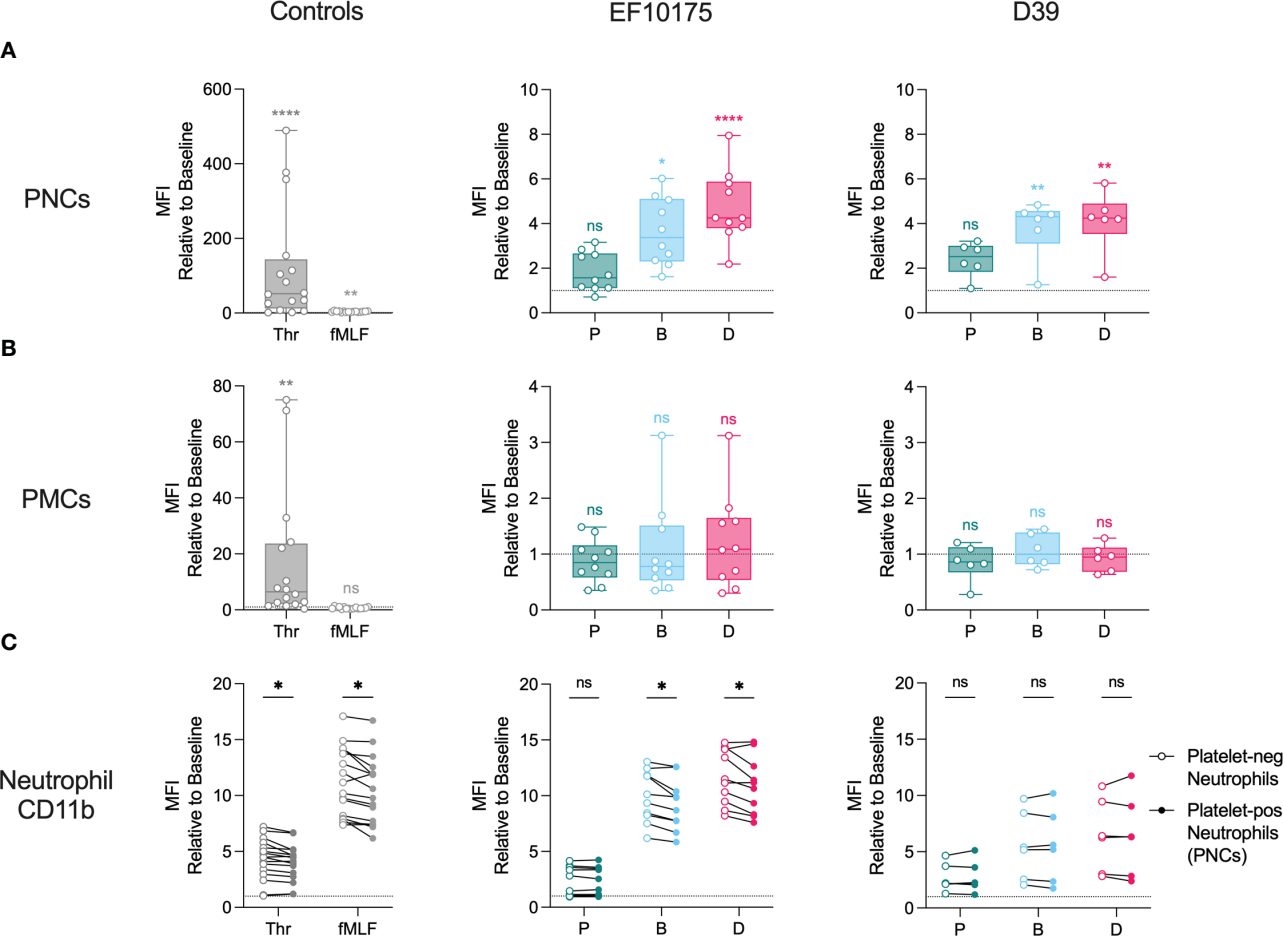
Formation of PNCs and PMCs in whole blood. Citrated whole blood was stimulated with HEPES (to determine baseline levels), 1 U/ml thrombin, 1 μM fMLF, or populations of planktonic (P), biofilm (B), or dispersed (D) pneumococci of strain EF10175 or strain D39 (1 x 10^7^ CFU/ml) for 15 min at 37°C. The median fluorescent intensity (MFI) of CD61 (platelet-positive events) on **(A)** neutrophils and **(B)** monocytes as well as **(C)** the MFI of activation marker CD11b on platelet-negative neutrophils and platelet-positive neutrophils were determined by flow cytometry. EF10175, n = 10 independent experiments from 8 healthy donors; D39, n = 6 independent experiments from 5 healthy donors. Data are shown relative to baseline, with the median ± interquartile range displayed in **(A**, **B)**. Statistical analysis was performed using Friedman test with Dunn’s multiple comparisons test or Wilcoxon matched-pairs signed rank test; ns, not significant; *P < 0.05, **P < 0.01, ****P < 0.0001.

As expected, the platelet agonist thrombin induced a robust and significant increase in PNC formation (**Figure 3A**) and PMC formation (**Figure 3B**) as compared to the baseline control (HEPES), whereas fMLF induced only low levels. Dispersed bacteria and biofilm bacteria from both pneumococcal strains induced significant PNC formation, while only a low and non-significant increase in PNC formation was observed in response to planktonic bacteria (**Figure 3A**). Dispersed bacteria induced a higher fold increase in PNC formation as compared with biofilm bacteria (**Supplementary Table 3).** When investigating platelet interactions with monocytes, no significant increase was observed in response to any of the pneumococcal populations (**Figure 3B**). This could be due to higher baseline levels of PMC formation that was not observed for PNC formation (**Supplementary Figure 5**). Therefore, any PMC formation induced by bacterial populations would not be discernable above baseline in our whole blood assays. Consequently, PNC formation but not PMC formation was further investigated.

To investigate the contribution of platelet binding to the neutrophil activation status, we compared surface expression of CD11b on platelet-positive neutrophils (PNCs) and platelet-negative neutrophils. Levels of CD11b were significantly higher on platelet-free neutrophils following stimulation with platelet agonist thrombin, leukocyte agonist fMLF, as well as with dispersed bacteria and biofilm bacteria of strain EF10175 (**Figure 3C**). No significant differences were observed in response to planktonic bacteria of strain EF10175 or to any population of strain D39.

These results demonstrate platelet activation via PNC formation in blood by bacterial populations associated with the infectious process but not by conventional broth-grown bacteria. Moreover, the activation status of neutrophils was not further elevated in PNCs.

### 3.3 Platelet activation in platelet-rich plasma

**Figure 4:**
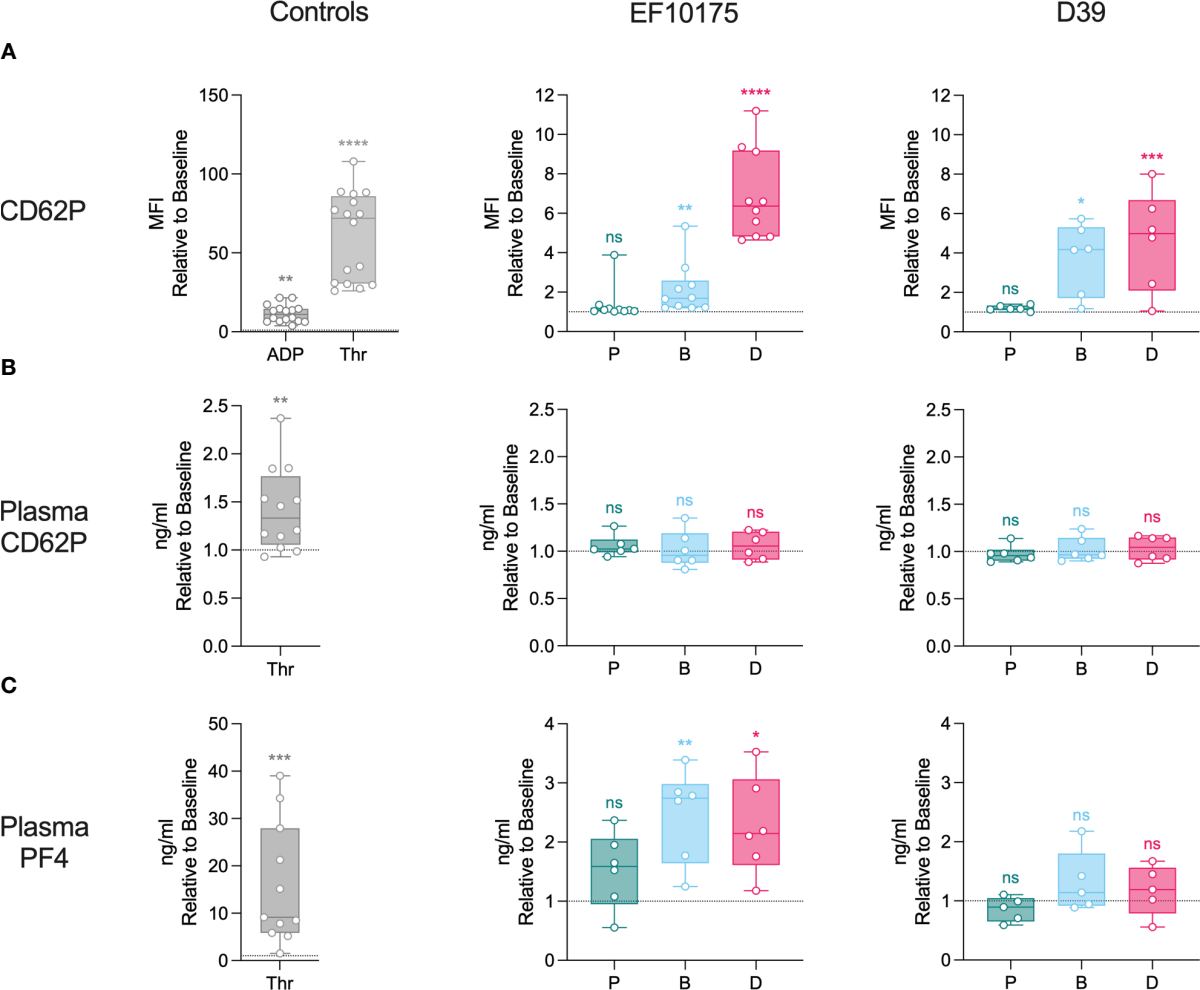
Platelet activation in platelet-rich plasma: α-granules. Diluted platelet-rich plasma was stimulated with HEPES (to determine baseline levels), 5 μM ADP, 1 U/ml thrombin, or populations of planktonic (P), biofilm (B), or dispersed (D) pneumococci of strain EF10175 or strain D39 (1 x 10^7^ CFU/ml) for 15 min at 37°C. **(A)** The median fluorescent intensity (MFI) of activation marker CD62P on platelets was determined by flow cytometry. Plasma levels of **(B)** soluble CD62P and **(C)** PF4 were determined by ELISA. Flow cytometry: EF10175, n = 10 independent experiments from 8 healthy donors; D39, n = 6 independent experiments from 5 healthy donors. ELISA: EF10175, n = 6 independent experiments from 6 healthy donors; D39, n = 6 independent experiments from 5 healthy donors. One replicate for strain D39 in **(C)** was excluded since the baseline was on average 40X lower than all other replicates. Data are shown relative to baseline with the median ± interquartile range displayed. Statistical analysis was performed using Wilcoxon matched-pairs signed rank test or Friedman test with Dunn’s multiple comparisons test; ns, not significant; *P < 0.05, **P < 0.01, ***P < 0.001, ****P < 0.0001.

**Figure 5:**
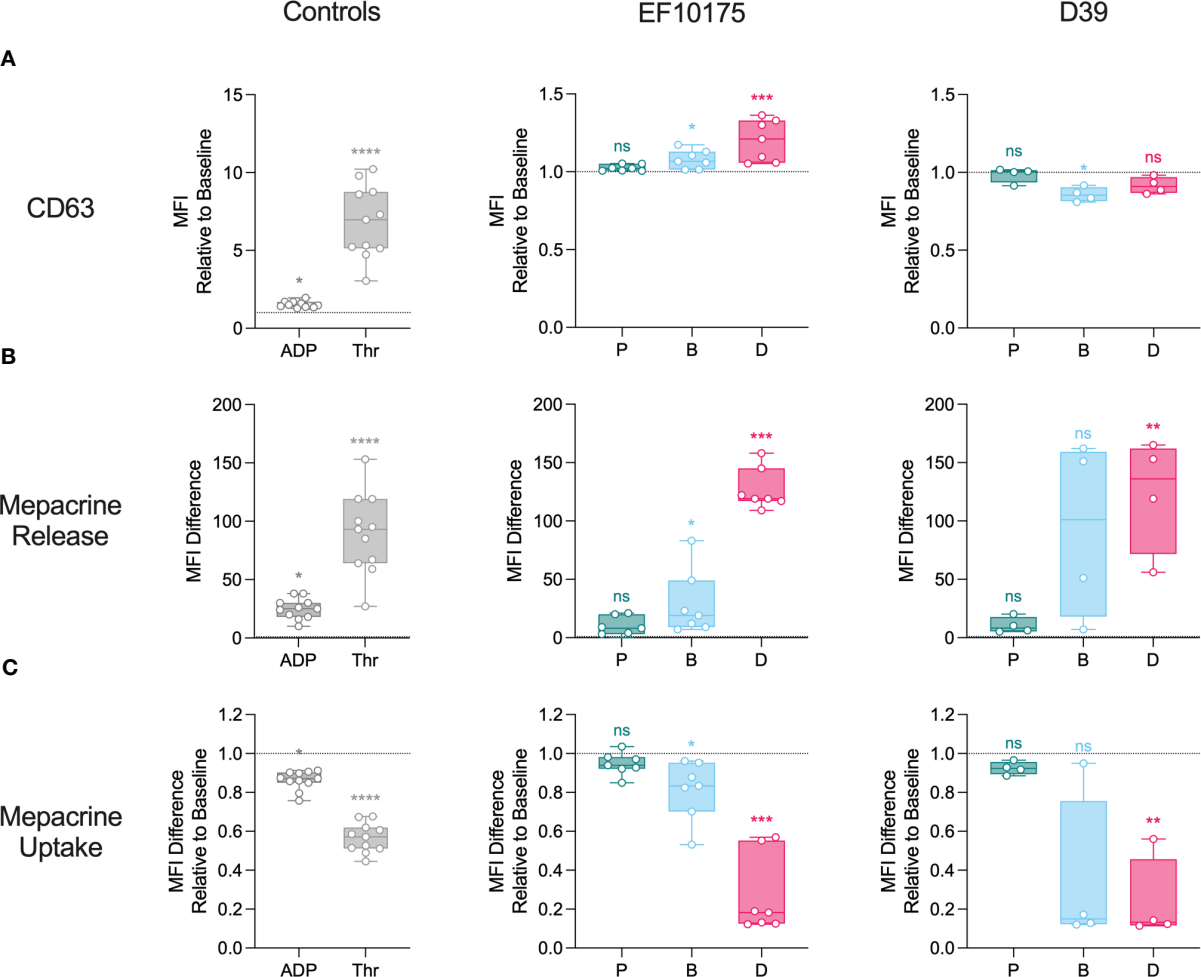
Platelet activation in platelet-rich plasma: dense granules. Diluted platelet-rich plasma was stimulated with HEPES (to determine baseline levels), 5 μM ADP, 1 U/ml thrombin, or populations of planktonic (P), biofilm (B), or dispersed (D) pneumococci of strain EF10175 or strain D39 (1 x 10^7^ CFU/ml) for 15 min at 37°C. **(A)** The median fluorescent intensity (MFI) of activation marker CD63 was determined by flow cytometry and is shown relative to baseline. **(B)** Mepacrine release (dense granule release) and **(C)** mepacrine uptake (residual dense granule content) were calculated as described in the Methods. EF10175, n = 7 independent experiments from 6 healthy donors; D39, n = 4 independent experiments from 3 healthy donors. Data are shown with the median ± interquartile range. Statistical analysis was performed using Friedman test with Dunn’s multiple comparisons test; ns, not significant; *P < 0.05, **P < 0.01, ***P < 0.001, ****P < 0.0001.

To further investigate platelet activation in an isolated platelet population, platelet-rich plasma (PRP) was stimulated *ex vivo* with platelet agonists, ADP and thrombin, or with pneumococcal populations of strains EF10175 and D39. Platelet activation was assessed by detection of α-granule markers (**Figure 4**) and dense granule markers (**Figure 5**). Platelet surface exposure of CD62P and CD63, mepacrine release, and mepacrine uptake were determined by flow cytometry. Plasma levels of soluble CD62P and PF4 were determined by ELISA.

There was a significant increase in CD62P on the platelet surface in response to ADP, thrombin, dispersed bacteria, and biofilm bacteria relative to the baseline control (HEPES), while planktonic bacteria failed to mediate any significant increase in CD62P (**Figure 4A**). As expected, platelet activation was more pronounced in response to the strong platelet agonist thrombin as compared to the weaker agonist ADP. The level of CD62P induced by dispersed bacteria of strain EF10175 was similar to that of ADP [median of 11.06 (IQR 6.70-14.57)] and the fold increase in CD62P was significantly higher than that induced by biofilm bacteria (**Supplementary Table 4**). Upon platelet activation, CD62P can be shed from the platelet surface, resulting in increased levels of soluble CD62P in plasma. However, this was not observed following stimulation with the pneumococcal populations under the experimental conditions used here (**Figure 4B**). Moreover, relatively low levels of soluble CD62P were observed with thrombin stimulation (~1.3-fold) in contrast to maximum levels obtained with 1% Triton X-100 [median of 5.62-fold (IQR 4.62-8.45)]. The duration of stimulation may not have allowed adequate cleavage of CD62P from the platelet surface, which may explain the discrepancy between surface and plasma levels of CD62P. Plasma levels of PF4, a marker of α-granule cargo release, increased significantly following stimulation with both thrombin as well as dispersed bacteria and biofilm bacteria of strain EF10175, while strain D39 failed to mediate significant PF4 release (**Figure 4C**).

Next, dense granule markers were investigated. Platelet agonists, ADP and thrombin, as well as dispersed bacteria and biofilm bacteria of strain EF10175 induced a significant increase in CD63 on the platelet surface as compared to the baseline control (HEPES) (**Figure 5A**). The level of CD63 induced by dispersed bacteria of strain EF10175 was similar to that of ADP [median of 1.47 (IQR 1.37-1.69)], and the fold increase in CD63 was significantly higher than that induced by biofilm bacteria (**Supplementary Table 4**). In contrast, populations of strain D39 did not induce CD63 levels above the baseline. Mepacrine release (as a measure of dense granule release) induced by exposure to strain EF10175 followed a similar pattern as observed with surface exposure of CD63 (**Figure 5B**; **Supplementary Table 4**). Both dispersed bacteria and biofilm bacteria from EF10175 induced mepacrine release, and the release induced by dispersed bacteria was significantly higher than that induced by biofilm bacteria (**Figure 5B**; **Supplementary Table 4** **).** Here, biofilm bacteria and dispersed bacteria of strain D39 also induced an increase in mepacrine release at a similar level as compared to strain EF10175. As expected, mepacrine uptake (as a measure of residual dense granule content) showed an inverse pattern where uptake of mepacrine in platelets was highest in the baseline control (HEPES) (**Figure 5C**; **Supplementary Table 4**). Stimulation with planktonic bacteria resulted in similar levels as baseline for all tested platelet activation markers.

**Figure 6:**
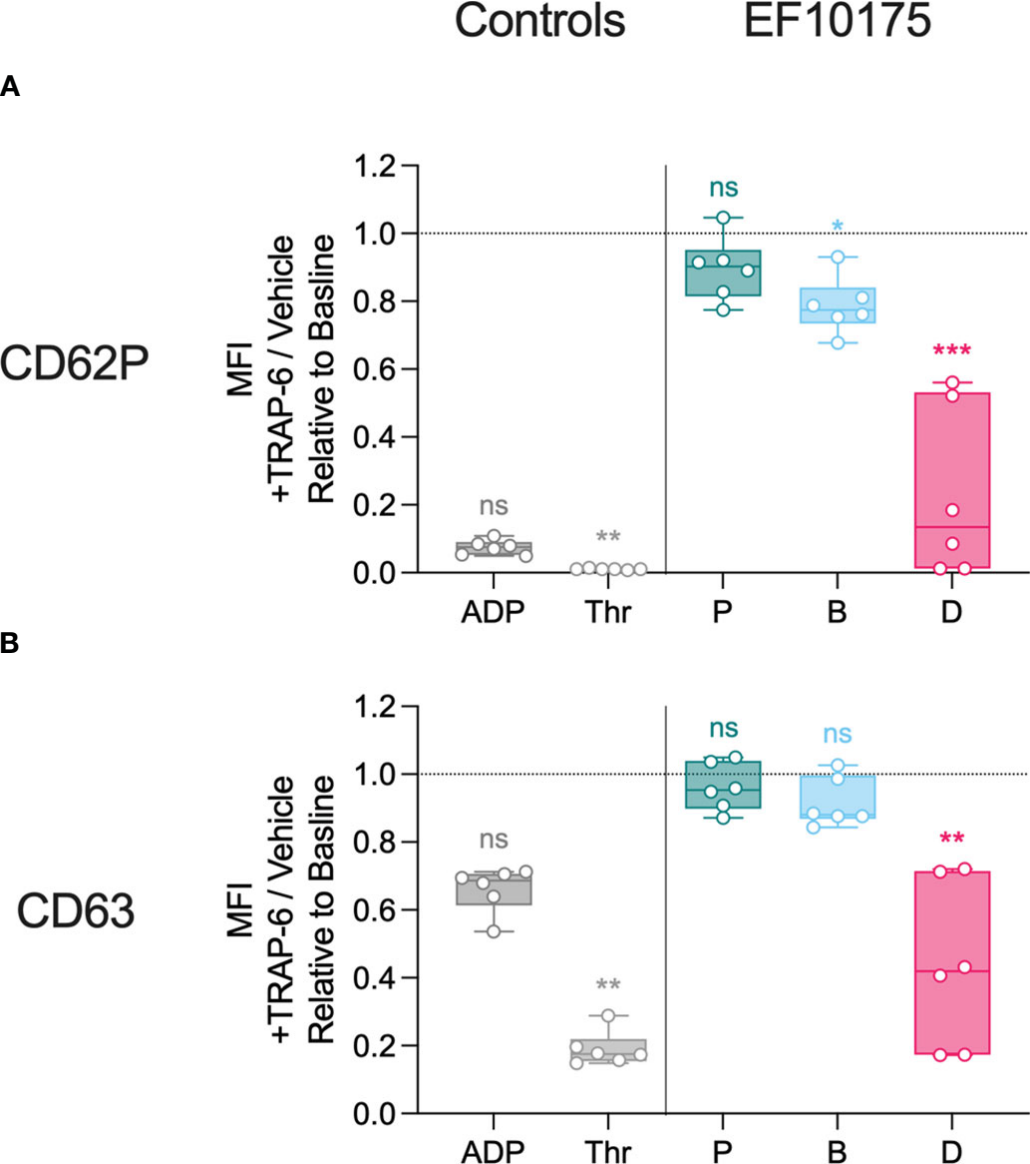
Platelet responsiveness to TRAP-6. Diluted platelet-rich plasma was stimulated with HEPES (to determine baseline levels), 5 μM ADP, 1 U/ml thrombin, or populations of planktonic (P), biofilm (B), or dispersed (D) pneumococci of strain EF10175 (1 x 10^7^ CFU/ml) for 15 min at 37°C and incubated for an additional 5 min at 37°C in the presence of 20 μM TRAP-6 or vehicle control. The median fluorescent intensity (MFI) of activation markers **(A)** CD62P and **(B)** CD63 on platelets were determined by flow cytometry and is calculated as the MFI of samples incubated with TRAP-6 divided by the MFI of samples incubated with vehicle control. n = 6 independent experiments from 6 healthy donors. Data are shown relative to baseline with the median ± interquartile range displayed. Statistical analysis was performed using Friedman test with Dunn’s multiple comparisons test; ns, not significant; *P < 0.05, **P < 0.01, ***P < 0.001.

Since dispersed bacteria of strain EF10175 induced the highest platelet activation, this strain was used to investigate platelet function post exposure to bacteria. After stimulation with platelet agonists or bacterial populations for 15 min, samples were subsequently stimulated with thrombin receptor activator peptide 6 (TRAP-6) or vehicle control for 5 min to determine the ability of platelets to respond to additional stimuli. Platelet surface expression of CD62P and CD63 were determined by flow cytometry. Additional response to TRAP-6 was calculated as the MFI ratio of samples stimulated with TRAP-6 to samples stimulated with vehicle control, and is shown relative to the baseline control (HEPES). Platelets stimulated with agonists or bacteria were responsive to subsequent TRAP-6 stimulation, and the degree of additional activation to TRAP-6 was inversely proportional to initial platelet activation to agonists or bacteria, i.e., lower initial activation resulted in higher activation in response to TRAP-6 (**Figure 6**). Platelet responsiveness post exposure to bacterial populations was in the range obtained after exposure to the strong platelet agonist thrombin (expected minimum level) and to HEPES (maximum level).

These results demonstrate that the distinct bacterial populations mediate differential platelet degranulation of α-granules and dense granules. Furthermore, subsequent platelet responsiveness to TRAP-6 was inversely related to the initial activation, suggesting that platelets remained functional after stimulation with bacterial populations.

### 3.4 Bacterial association with immune cells

Bacteria can stimulate immune cells by direct association or indirectly via released products. To examine direct bacterial association with the immune cells investigated in our experimental setup, pneumococcal populations of EF10175 were stained with OG488-X and used in *ex vivo* stimulations of whole blood and PRP. Flow cytometry was used to determine bacteria-neutrophil associates, bacteria-monocyte associates, and bacteria-platelet associates by measuring OG488-X staining on neutrophils, monocytes, and platelets, respectively.

**Figure 7:**
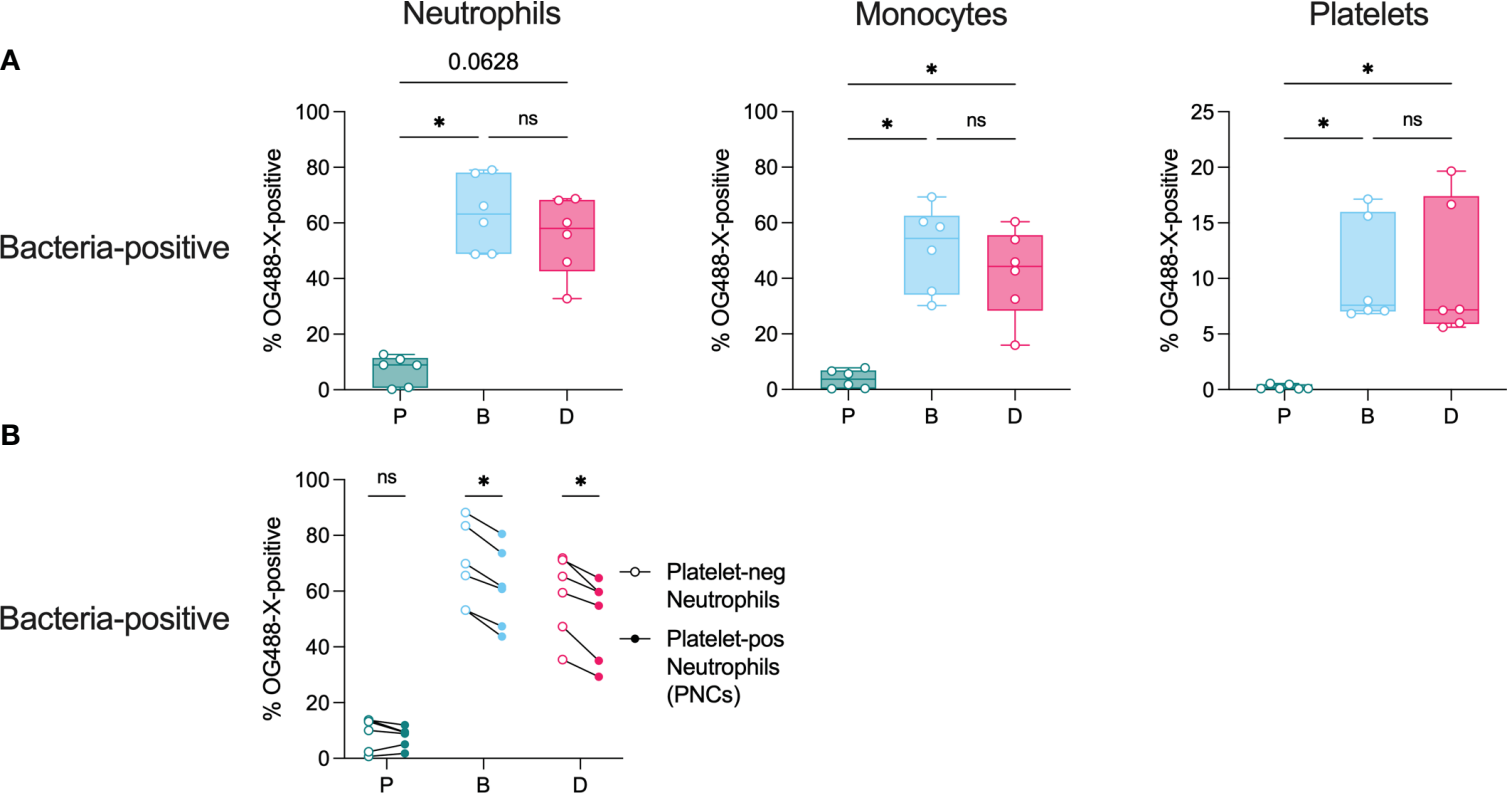
Bacterial association with immune cells. Citrated whole blood and diluted platelet-rich plasma (PRP) were stimulated with non-stained or OG488-X-stained populations of planktonic (P), biofilm (B), or dispersed (D) pneumococci of strain EF10175 (1 x 10^7^ CFU/ml) for 15 min at 37°C. The percentage of FITC-positive **(A)** neutrophils, monocytes, and platelets as well as **(B)** platelet-negative neutrophils and platelet-positive neutrophils were determined by flow cytometry. Bacteria association was calculated as the difference between the percentages of samples stimulated with OG488-X-stained bacteria and non-stained bacteria. Whole blood, n = 6 independent experiments from 5 healthy donors; PRP, n = 6 independent experiments from 6 healthy donors. Data in **(A)** are shown with the median ± interquartile range. Statistical analysis was performed using Friedman test with Dunn’s multiple comparisons test or Wilcoxon matched-pairs signed rank test; ns, not significant; *P < 0.05.

Biofilm bacteria and dispersed bacteria were associated with a higher proportion of neutrophils (~7-fold) and monocytes (~13-fold) as compared with planktonic bacteria (**Figure 7A**). Interestingly, there were no significant differences in immune cell association between the biofilm and dispersed populations, although there was a trend towards higher cell association for biofilm bacteria. Furthermore, for neutrophils, significantly more biofilm bacteria and dispersed bacteria were associated with platelet-free neutrophils than with platelet-bound neutrophils (PNCs) (**Figure 7B**). In PRP, biofilm bacteria and dispersed bacteria were also more associated with platelets (~7-fold) as compared with planktonic bacteria (**Figure 7A**), which was further increased after subsequent stimulation with TRAP-6 (**Supplementary Figure 6**).

These results demonstrate that among the bacterial populations investigated, biofilm-derived bacteria associate significantly more with neutrophils, monocytes, and platelets. Moreover, the presence of platelets did not further increase bacterial association with neutrophils.

## 4 Discussion

Biofilm-grown bacteria and bacteria that are released from biofilms in response to changes in the local environment (i.e., dispersed bacteria) represent populations associated with distinct phenotypes of bacteria during pneumococcal colonization and transition to disease, respectively (Marks et al., 2013; Pettigrew et al., 2014). Herein, complementary assays were used to investigate the activation of innate immune cells by pneumococcal populations associated with distinct stages of the infectious process as well as conventional broth-grown culture.

In general, pneumococci associated with the infectious process (biofilm bacteria and dispersed bacteria) induced higher levels of activation of neutrophils, monocytes, and platelets than observed after exposure to conventional broth-grown planktonic bacteria. We observed upregulation of neutrophil and monocyte activation markers, such as CD11b on the surface of cells as well as MPO in plasma. Platelet activation was evident both by the formation of PNCs in whole blood and by platelet degranulation of α-granules (CD62P, PF4) and dense granules (CD63, mepacrine) in PRP. Furthermore, dispersed bacteria induced more activation than biofilm bacteria for the majority of activation markers, although the differences were not as distinct as hypothesized. Higher bacterial association with immune cells was observed with biofilm-derived bacteria as compared with planktonic bacteria. Dispersed bacteria induced higher activation despite lower bacterial association as compared with biofilm bacteria, suggesting that dispersed bacteria may cause even more activation that may occur through a mechanism separate from direct association with the immune cells. We also observed some variations in immune activation between the two investigated strains. In support of this, the degree of platelet activation has been reported to vary across pneumococcal strains (Keane et al., 2010; de Stoppelaar et al., 2016). Despite some observed differences between strains, the general trends were similar in that biofilm-derived bacteria induced higher activation than planktonic bacteria.

Unlike other pathogens, such as *Streptococcus pyogenes* and *Staphylococcus aureus*, that produce a variety of tissue-damaging substances, pneumococci produce relatively few mediators of cytotoxicity, with pneumolysin being the major one. Rather, intense inflammation is the hallmark of pneumococcal disease (Loughran et al., 2019). Immune cell activation can lead to recruitment and activation of other immune cells, amplifying the inflammatory response. However, a dysregulated immune response can be detrimental. For example, neutrophils play a complex role in pneumococcal disease as they contribute to immunity and can also contribute to excessive inflammation and tissue damage (Pechous, 2017; Domon and Terao, 2021). Dysregulated platelet activation can lead to increased platelet consumption and depletion, resulting in thrombocytopenia that is often observed during systemic infection (Page and Pretorius, 2020). Thus, the observed elevated activation by biofilm-derived bacteria may facilitate clearance of the bacteria but may also lead to collateral damage from excessive inflammation that is often observed during invasive pneumococcal disease.

Activated platelets play a role in inflammation and antimicrobial defense. Platelets can interact with neutrophils and modulate their function. However, we did not observe a substantial contribution of a platelets to either neutrophil activation status or bacterial association with neutrophils. Instead, platelet-free neutrophils exhibited significantly higher levels of both CD11b surface expression and bacteria-neutrophil associates in response to biofilm-derived bacteria. The tendency for more CD11b on platelet-free neutrophils has also been observed in response to oral bacteria (Nicu et al., 2009). Isolated platelets and platelet releasates have been reported to kill *Staphylococcus aureus* (Wolff et al., 2020). However, this antimicrobial activity is not universal, as bactericidal effects have not been observed against group B *Streptococcus* (Uchiyama et al., 2019) or pneumococci (Wolff et al., 2020). We also did not observe killing of pneumococci by either unstimulated or thrombin-activated platelets and respective releasates in our assays. Previously, pneumococci have been shown to render platelets non-responsive to the platelet agonist TRAP-6 (Wolff et al., 2020). In contrast, we observed that platelets remained functional after stimulation with pneumococcal populations, maintaining responsiveness to subsequent TRAP-6 stimulation at levels comparable to physiological agonists. We demonstrate that platelet responsiveness to TRAP-6 occurs in a manner inversely related to the initial activation by agonists or bacteria. The apparent discrepancy in TRAP-6 responsiveness may be due to the use of encapsulated strains in our study and a capsule mutant of strain in the previous study. Unencapsulated strains have been reported to activate platelets to a greater extent than encapsulated strains (Keane et al., 2010; de Stoppelaar et al., 2016). Therefore, a higher initial activation induced by the capsule mutant strain would result in a lower capacity for subsequent activation to TRAP-6. 

The overall lack of immune cell activation by planktonic bacteria may relate to the low association that these bacteria exhibited with the immune cells. However, bacterial attachment does not always predict the activation response of host cells. For instance, in a previous study on human respiratory epithelial cells, biofilm bacteria adhered the most as compared to dispersed bacteria and planktonic bacteria, yet the highest cytokine levels were induced by dispersed bacteria (Marks et al., 2013). An alternative explanation could be that biofilm-derived bacteria retain some components of the biofilm matrix. Lysis of pneumococcal cells is crucial for pneumococcal biofilm formation (Marks et al., 2012), indicating that materials from lysed pneumococci contribute to the formation of the biofilm matrix. Therefore, remnants of the biofilm matrix may serve as recognizable pattern associated molecular patterns (PAMPs) for immune cells. The presence of biofilm matrix may accurately represent the *in vivo* situation, as pneumococci form biofilms during colonization (Marks et al., 2012), and colonization precedes disease (Bogaert et al., 2004; Short and Diavatopoulos, 2015).

The distinct expression profiles of the different pneumococcal populations may contribute to the different immune activation phenotypes observed in this study, as the activation of leukocytes as well as platelets by pneumococci may involve interactions with the bacteria or via bacterial products (Keane et al., 2010; Arman et al., 2014; de Stoppelaar et al., 2016; Anderson and Feldman, 2017; Jahn et al., 2022b). For example, *nanA* (neuraminidase) and *ply* (pneumolysin) are upregulated in dispersed pneumococci as compared with biofilm bacteria in transcriptional studies (Pettigrew et al., 2014). Platelet desialylation by pneumococcal NanA leads to platelet hyperreactivity (Kullaya et al., 2018). Furthermore, platelet desialylation occurs during pneumococcal infection, increasing the clearance of platelets (Grewal et al., 2013). These mechanisms may contribute to thrombocytopenia that is observed during sepsis. In recent years, there has been growing evidence of a role for pneumolysin in platelet activation and aggregation (Nel et al., 2017; Letsiou et al., 2021) via pore formation and loss of platelet function (Jahn et al., 2020; Wiebe et al., 2022; Jahn et al., 2022a). We have not addressed the role of bacterial products in the present study. Although, since pneumolysin is required for optimal pneumococcal biofilm formation (Marks et al., 2012), studying pneumolysin in the context of biofilm-derived populations is challenging.

Differential modulation of immune responses by planktonic-grown bacteria and biofilm-grown bacteria or respective bacterial products has been observed in species where pathogenicity relies on biofilm formation (Oscarsson et al., 2008; Ding and Tan, 2016; Guilhen et al., 2019; Kaya et al., 2021; Seebach et al., 2023). Variation in host responses depending on the species and the mode of growth suggests different strategies by bacteria to persist in the host. The mode of growth of the bacteria is an important factor to consider in experimental design. Studying planktonic bacteria remains valuable for understanding basic bacterial physiology and standardizing research. However, the incorporation of bacterial populations adapted to different host niches that may potentially better reflect the conditions encountered during the infectious process is increasingly important.

Our findings provide strong support for different responses induced by niche-distinct pneumococcal populations, especially between bacterial populations associated with the infectious process and conventional broth-grown planktonic bacteria. This study highlights bacterial population differences in the activation of and association with immune cells, emphasizing the importance of considering bacterial growth conditions in research on host-pathogen interactions, especially for pathogens that have a biofilm (and triggered dispersal) state. A focus on distinct bacterial populations associated with stages of disease development will move forward the understanding of host-pathogen interactions and may help identify novel immunomodulatory targets to interfere with the infectious process.

## Data availability statement

The raw data supporting the conclusions of this article will be made available by the authors, without undue reservation.

## Ethics statement

The studies involving humans were approved by The Regional Ethical Review Authority, Lund. The studies were conducted in accordance with the local legislation and institutional requirements. The participants provided their written informed consent to participate in this study.

## Author contributions

YC: Conceptualization, Formal analysis, Funding acquisition, Investigation, Methodology, Project administration, Visualization, Writing – original draft, Writing – review & editing. MM: Investigation, Writing – review & editing. AH: Conceptualization, Funding acquisition, Resources, Writing – review & editing. OS: Conceptualization, Funding acquisition, Methodology, Project administration, Resources, Supervision, Writing – original draft, Writing – review & editing.

## References

[B1] Anderson R. Feldman C. (2017). Review manuscript: Mechanisms of platelet activation by the pneumococcus and the role of platelets in community-acquired pneumonia. J. Infect. 75, 473–485. doi: 10.1016/j.jinf.2017.09.013 28943342

[B2] Andersson B. Dahmen J. Frejd T. Leffler H. Magnusson G. Noori G.. (1983). Identification of an active disaccharide unit of a glycoconjugate receptor for pneumococci attaching to human pharyngeal epithelial cells. J. Exp. Med. 158, 559–570. doi: 10.1084/jem.158.2.559 6886624 PMC2187347

[B3] Arman M. Krauel K. Tilley D. O. Weber C. Cox D. Greinacher A.. (2014). Amplification of bacteria-induced platelet activation is triggered by FcgammaRIIA, integrin alphaIIbbeta3, and platelet factor 4. Blood 123, 3166–3174. doi: 10.1182/blood-2013-11-540526 24642751 PMC4023422

[B4] Avery O. T. Macleod C. M. McCarty M. (1944). Studies on the chemical nature of the substance inducing transformation of pneumococcal types: induction of transformation by a desoxyribonucleic acid fraction isolated from pneumococcus type iii. J. Exp. Med. 79, 137–158. doi: 10.1084/jem.79.2.137 19871359 PMC2135445

[B5] Bakaletz L. O. (2017). Viral-bacterial co-infections in the respiratory tract. Curr. Opin. Microbiol. 35, 30–35. doi: 10.1016/j.mib.2016.11.003 27940028 PMC7108227

[B6] Berger G. Hartwell D. W. Wagner D. D. (1998). P-Selectin and platelet clearance. Blood 92, 4446–4452. doi: 10.1182/blood.V92.11.4446 9834252

[B7] Blanchette-Cain K. Hinojosa C. A. Akula Suresh Babu R. Lizcano A. Gonzalez-Juarbe N. Munoz-Almagro C.. (2013). Streptococcus pneumoniae biofilm formation is strain dependent, multifactorial, and associated with reduced invasiveness and immunoreactivity during colonization. mBio 4, e00745–e00713. doi: 10.1128/mBio.00745-13 24129258 PMC3812715

[B8] Bogaert D. De Groot R. Hermans P. W. (2004). Streptococcus pneumoniae colonization: the key to pneumococcal disease. Lancet Infect. Dis. 4, 144–154. doi: 10.1016/S1473-3099(04)00938-7 14998500

[B9] Bosch A. A. Biesbroek G. Trzcinski K. Sanders E. A. Bogaert D. (2013). Viral and bacterial interactions in the upper respiratory tract. PloS Pathog. 9, e1003057. doi: 10.1371/journal.ppat.1003057 23326226 PMC3542149

[B10] Chao Y. Bergenfelz C. Hakansson A. P. (2019). Growing and characterizing biofilms formed by streptococcus pneumoniae. Methods Mol. Biol. 1968, 147–171. doi: 10.1007/978-1-4939-9199-0_13 30929213

[B11] Chao Y. Bergenfelz C. Sun R. Han X. Achour A. Hakansson A. P. (2020). The serine protease HtrA plays a key role in heat-induced dispersal of pneumococcal biofilms. Sci. Rep. 10, 22455. doi: 10.1038/s41598-020-80233-0 33384455 PMC7775458

[B12] Chao Y. Rebetz J. Blackberg A. Hovold G. Sunnerhagen T. Rasmussen M.. (2021). Distinct phenotypes of platelet, monocyte, and neutrophil activation occur during the acute and convalescent phase of COVID-19. Platelets 32, 1092–1102. doi: 10.1080/09537104.2021.1921721 33999778 PMC8146300

[B13] de Stoppelaar S. F. Claushuis T. A. Schaap M. C. Hou B. van der Poll T. Nieuwland R.. (2016). Toll-like receptor signaling is not involved in platelet response to streptococcus pneumoniae *in vitro* or *in vivo* . PloS One 11, e0156977. doi: 10.1371/journal.pone.0156977 27253707 PMC4890788

[B14] Ding Q. Tan K. S. (2016). The danger signal extracellular ATP is an inducer of fusobacterium nucleatum biofilm dispersal. Front. Cell Infect. Microbiol. 6. doi: 10.3389/fcimb.2016.00155 PMC5112537 27909688

[B15] Domon H. Terao Y. (2021). The role of neutrophils and neutrophil elastase in pneumococcal pneumonia. Front. Cell Infect. Microbiol. 11. doi: 10.3389/fcimb.2021.615959 PMC8008068 33796475

[B16] Flemming H. C. Wingender J. Szewzyk U. Steinberg P. Rice S. A. Kjelleberg S. (2016). Biofilms: an emergent form of bacterial life. Nat. Rev. Microbiol. 14, 563–575. doi: 10.1038/nrmicro.2016.94 27510863

[B17] Grewal P. K. Aziz P. V. Uchiyama S. Rubio G. R. Lardone R. D. Le D.. (2013). Inducing host protection in pneumococcal sepsis by preactivation of the Ashwell-Morell receptor. Proc. Natl. Acad. Sci. U.S.A. 110, 20218–20223. doi: 10.1073/pnas.1313905110 24284176 PMC3864324

[B18] Guilhen C. Miquel S. Charbonnel N. Joseph L. Carrier G. Forestier C.. (2019). Colonization and immune modulation properties of Klebsiella pneumoniae biofilm-dispersed cells. NPJ Biofilms Microbiomes 5, 25. doi: 10.1038/s41522-019-0098-1 31583108 PMC6760147

[B19] Hall-Stoodley L. Hu F. Z. Gieseke A. Nistico L. Nguyen D. Hayes J.. (2006). Direct detection of bacterial biofilms on the middle-ear mucosa of children with chronic otitis media. JAMA 296, 202–211. doi: 10.1001/jama.296.2.202 16835426 PMC1885379

[B20] Heijnen H. van der Sluijs P. (2015). Platelet secretory behavior: as diverse as the granules … or not? J. Thromb. Haemost. 13, 2141–2151. doi: 10.1111/jth.13147 26391322

[B21] Hoa M. Tomovic S. Nistico L. Hall-Stoodley L. Stoodley P. Sachdeva L.. (2009). Identification of adenoid biofilms with middle ear pathogens in otitis-prone children utilizing SEM and FISH. Int. J. Pediatr. Otorhinolaryngol 73, 1242–1248. doi: 10.1016/j.ijporl.2009.05.016 19525016

[B22] Irvin J. L. Irvin E. M. (1954). The interaction of quinacrine with adenine nucleotides. J. Biol. Chem. 210, 45–56. doi: 10.1016/S0021-9258(18)65431-6 13201568

[B23] Jahn K. Handtke S. Palankar R. Kohler T. P. Wesche J. Wolff M.. (2022a). alpha-hemolysin of Staphylococcus aureus impairs thrombus formation. J. Thromb. Haemost. 20, 1464–1475. doi: 10.1111/jth.15703 35303391

[B24] Jahn K. Handtke S. Palankar R. Weissmuller S. Nouailles G. Kohler T. P.. (2020). Pneumolysin induces platelet destruction, not platelet activation, which can be prevented by immunoglobulin preparations *in vitro* . Blood Adv. 4, 6315–6326. doi: 10.1182/bloodadvances.2020002372 33351126 PMC7756997

[B25] Jahn K. Kohler T. P. Swiatek L. S. Wiebe S. Hammerschmidt S. (2022b). Platelets, bacterial adhesins and the pneumococcus. Cells 11, 1121. doi: 10.3390/cells11071121 35406684 PMC8997422

[B26] Jusot J. F. Neill D. R. Waters E. M. Bangert M. Collins M. Bricio Moreno L.. (2017). Airborne dust and high temperatures are risk factors for invasive bacterial disease. J. Allergy Clin. Immunol. 139, 977–986.e972. doi: 10.1016/j.jaci.2016.04.062 27523432 PMC5338876

[B27] Kapur R. Zufferey A. Boilard E. Semple J. W. (2015). Nouvelle cuisine: platelets served with inflammation. J. Immunol. 194, 5579–5587. doi: 10.4049/jimmunol.1500259 26048965

[B28] Kaya E. Batoni G. Di Luca M. Apolloni E. Mazzoni A. Maisetta G.. (2021). Planktonic and Biofilm-Associated Pseudomonas aeruginosa and Staphylococcus epidermidis Elicit Differential Human Peripheral Blood Cell Responses. Microorganisms 9, 1846. doi: 10.3390/microorganisms9091846 34576742 PMC8470397

[B29] Keane C. Tilley D. Cunningham A. Smolenski A. Kadioglu A. Cox D.. (2010). Invasive Streptococcus pneumoniae trigger platelet activation via Toll-like receptor 2. J. Thromb. Haemost. 8, 2757–2765. doi: 10.1111/j.1538-7836.2010.04093.x 20946179

[B30] Kullaya V. de Jonge M. I. Langereis J. D. van der Gaast-de Jongh C. E. Bull C. Adema G. J.. (2018). Desialylation of platelets by pneumococcal neuraminidase A induces ADP-dependent platelet hyperreactivity. Infect. Immun. 86, e00213–18. doi: 10.1128/IAI.00213-18 PMC6204724 30037798

[B31] Letsiou E. Teixeira Alves L. G. Felten M. Mitchell T. J. Muller-Redetzky H. C. Dudek S. M.. (2021). Neutrophil-derived extracellular vesicles activate platelets after pneumolysin exposure. Cells 10, 3581. doi: 10.3390/cells10123581 34944089 PMC8700313

[B32] Li Z. Delaney M. K. O’Brien K. A. Du X. (2010). Signaling during platelet adhesion and activation. Arterioscler. Thromb. Vasc. Biol. 30, 2341–2349. doi: 10.1161/ATVBAHA.110.207522 21071698 PMC3085271

[B33] Loughran A. J. Orihuela C. J. Tuomanen E. I. (2019). Streptococcus pneumoniae: invasion and inflammation. Microbiol. Spectr. 7. doi: 10.1128/microbiolspec.GPP3-0004-2018 PMC6422050 30873934

[B34] Marks L. R. Davidson B. A. Knight P. R. Hakansson A. P. (2013). Interkingdom signaling induces Streptococcus pneumoniae biofilm dispersion and transition from asymptomatic colonization to disease. mBio 4, e00438–13. doi: 10.1128/mBio.00438-13 PMC3735180 23882016

[B35] Marks L. R. Parameswaran G. I. Hakansson A. P. (2012). Pneumococcal interactions with epithelial cells are crucial for optimal biofilm formation and colonization *in vitro* and *in vivo* . Infect. Immun. 80, 2744–2760. doi: 10.1128/IAI.00488-12 22645283 PMC3434590

[B36] Michelson A. D. Barnard M. R. Hechtman H. B. MacGregor H. Connolly R. J. Loscalzo J.. (1996). *In vivo* tracking of platelets: circulating degranulated platelets rapidly lose surface P-selectin but continue to circulate and function. Proc. Natl. Acad. Sci. U.S.A. 93, 11877–11882. doi: 10.1073/pnas.93.21.11877 8876231 PMC38152

[B37] Munoz-Elias E. J. Marcano J. Camilli A. (2008). Isolation of Streptococcus pneumoniae biofilm mutants and their characterization during nasopharyngeal colonization. Infect. Immun. 76, 5049–5061. doi: 10.1128/IAI.00425-08 18794289 PMC2573321

[B38] Nel J. G. Durandt C. Theron A. J. Tintinger G. R. Pool R. Richards G. A.. (2017). Pneumolysin mediates heterotypic aggregation of neutrophils and platelets *in vitro* . J. Infect. 74, 599–608. doi: 10.1016/j.jinf.2017.02.010 28267572

[B39] Nicu E. A. van der Velden U. Nieuwland R. Everts V. Loos B. G. (2009). Elevated platelet and leukocyte response to oral bacteria in periodontitis. J. Thromb. Haemost. 7, 162–170. doi: 10.1111/j.1538-7836.2008.03219.x 18983491

[B40] O’Brien K. L. Wolfson L. J. Watt J. P. Henkle E. Deloria-Knoll M. McCall N.. (2009). Burden of disease caused by Streptococcus pneumoniae in children younger than 5 years: global estimates. Lancet 374, 893–902. doi: 10.1016/S0140-6736(09)61204-6 19748398

[B41] Oscarsson J. Karched M. Thay B. Chen C. Asikainen S. (2008). Proinflammatory effect in whole blood by free soluble bacterial components released from planktonic and biofilm cells. BMC Microbiol. 8, 206. doi: 10.1186/1471-2180-8-206 19038023 PMC2612679

[B42] Page M. J. Pretorius E. (2020). A champion of host defense: A generic large-scale cause for platelet dysfunction and depletion in infection. Semin. Thromb. Hemost 46, 302–319. doi: 10.1055/s-0040-1708827 32279287 PMC7339151

[B43] Palm F. Chowdhury S. Wettemark S. Malmstrom J. Happonen L. Shannon O. (2022). Distinct serotypes of streptococcal M proteins mediate fibrinogen-dependent platelet activation and proinflammatory effects. Infect. Immun. 90, e0046221. doi: 10.1128/iai.00462-21 34898252 PMC8852700

[B44] Pechous R. D. (2017). With friends like these: the complex role of neutrophils in the progression of severe pneumonia. Front. Cell Infect. Microbiol. 7. doi: 10.3389/fcimb.2017.00160 PMC5410563 28507954

[B45] Pettigrew M. M. Marks L. R. Kong Y. Gent J. F. Roche-Hakansson H. Hakansson A. P. (2014). Dynamic changes in the Streptococcus pneumoniae transcriptome during transition from biofilm formation to invasive disease upon influenza A virus infection. Infect. Immun. 82, 4607–4619. doi: 10.1128/IAI.02225-14 25135685 PMC4249342

[B46] Rossaint J. Zarbock A. (2015). Platelets in leucocyte recruitment and function. Cardiovasc. Res. 107, 386–395. doi: 10.1093/cvr/cvv048 25712962

[B47] Schrottmaier W. C. Kral-Pointner J. B. Salzmann M. Mussbacher M. Schmuckenschlager A. Pirabe A.. (2022). Platelet p110beta mediates platelet-leukocyte interaction and curtails bacterial dissemination in pneumococcal pneumonia. Cell Rep. 41, 111614. doi: 10.1016/j.celrep.2022.111614 36351402

[B48] Seebach E. Sonnenmoser G. Kubatzky K. F. (2023). Staphylococcus aureus planktonic but not biofilm environment induces an IFN-beta macrophage immune response via the STING/IRF3 pathway. Virulence 14, 2254599. doi: 10.1080/21505594.2023.2254599 37655977 PMC10496530

[B49] Sharda A. Flaumenhaft R. (2018). The life cycle of platelet granules. F1000Res 7, 236. doi: 10.12688/f1000research.13283.1 29560259 PMC5832915

[B50] Short K. R. Diavatopoulos D. A. (2015). “Nasopharyngeal colonization with Streptococcus pneumoniae,” in Streptococcus pneumoniae: molecular mechanisms of host-pathogen interactions. Eds. Brown J. Hammerschmidt S. Orihuela C. J. (Academic Press, London), 279–291.

[B51] Short K. R. Reading P. C. Wang N. Diavatopoulos D. A. Wijburg O. L. (2012). Increased nasopharyngeal bacterial titers and local inflammation facilitate transmission of Streptococcus pneumoniae. mBio 3, e00255–12. doi: 10.1128/mBio.00255-12 PMC3518912 23015738

[B52] Stahl A. L. Sartz L. Nelsson A. Bekassy Z. D. Karpman D. (2009). Shiga toxin and lipopolysaccharide induce platelet-leukocyte aggregates and tissue factor release, a thrombotic mechanism in hemolytic uremic syndrome. PloS One 4, e6990. doi: 10.1371/journal.pone.0006990 19750223 PMC2735777

[B53] Teo S. M. Mok D. Pham K. Kusel M. Serralha M. Troy N.. (2015). The infant nasopharyngeal microbiome impacts severity of lower respiratory infection and risk of asthma development. Cell Host Microbe 17, 704–715. doi: 10.1016/j.chom.2015.03.008 25865368 PMC4433433

[B54] Uchiyama S. Sun J. Fukahori K. Ando N. Wu M. Schwarz F.. (2019). Dual actions of group B Streptococcus capsular sialic acid provide resistance to platelet-mediated antimicrobial killing. Proc. Natl. Acad. Sci. U.S.A. 116, 7465–7470. doi: 10.1073/pnas.1815572116 30910970 PMC6462088

[B55] van den Boogaard F. E. Schouten M. de Stoppelaar S. F. Roelofs J. J. Brands X. Schultz M. J.. (2015). Thrombocytopenia impairs host defense during murine Streptococcus pneumoniae pneumonia. Crit. Care Med. 43, e75–e83. doi: 10.1097/CCM.0000000000000853 25627210

[B56] Wall J. E. Buijs-Wilts M. Arnold J. T. Wang W. White M. M. Jennings L. K.. (1995). A flow cytometric assay using mepacrine for study of uptake and release of platelet dense granule contents. Br. J. Haematol 89, 380–385. doi: 10.1111/j.1365-2141.1995.tb03315.x 7873389

[B57] Wiebe F. Handtke S. Wesche J. Schnarre A. Palankar R. Wolff M.. (2022). Polyvalent immunoglobulin preparations inhibit pneumolysin-induced platelet destruction. Thromb. Haemost. 122, 1147–1158. doi: 10.1055/a-1723-1880 34918314 PMC9385248

[B58] Wolff M. Handtke S. Palankar R. Wesche J. Kohler T. P. Kohler C.. (2020). Activated platelets kill Staphylococcus aureus, but not Streptococcus pneumoniae-The role of FcgammaRIIa and platelet factor 4/heparinantibodies. J. Thromb. Haemost. 18, 1459–1468. doi: 10.1111/jth.14814 32237268

