# Supplementary material for: Biofilm-dispersed pneumococci induce elevated leukocyte and platelet activation
Source: Front Cell Infect Microbiol. 2024 Aug 1;14:1405333. doi: 10.3389/fcimb.2024.1405333 (PMC11324597; doi:10.3389/fcimb.2024.1405333)
Supplement: Supplementary file 1 [file DataSheet_1.pdf]

Supplementary Material

1 Supplementary Figures and Tables

1.1 Supplementary Tables

Supplementary Table 1. Biofilm and biofilm dispersal quantification

	Biofilm		Biofilm dispersal
Strain	Biomass<br>Log_{10} CFU/ml	Gentamicin sensitivity<br>Log_{10} CFU/ml	Ratio CFU/ml at 38.5°C / 34°C
EF10175	8.01 (7.84-8.13)	0.87 (0.70-1.06)	2.21 (2.01-2.59)
D39	8.42 (8.24-8.44)	0.14 (0.11-0.32)	2.75 (2.72-3.05)

Values shown as median (IQR)

Supplementary Table 2. Strain EF10175 viability was comparable with or without OG488-X staining

Population	Vehicle<br>Log_{10} CFU/ml [a]	OG488-X<br>Log_{10} CFU/ml [a]	*P* value [b]
Planktonic	7.94 (7.90-8.10)	8.10 (7.94-8.15)	0.50, ns
Biofilm	8.12 (8.10-8.14)	8.01 (8.00-8-05)	0.25, ns
Dispersed	8.03 (7.97-8.06)	8.06 (7.98-8.12)	0.50, ns

[a] Values shown as median (IQR)

[b] Wilcoxon matched-pairs signed rank test

Supplementary Table 3. Relative fold increase by dispersed bacteria compared with biofilm bacteria in whole blood

Marker	Strain	Dispersed/Biofilm [a]	*P* value [b]
Neutrophil CD11b	EF10175	1.21 (1.14-1.25)	< 0.01, **
	D39	1.17 (1.13-1.27)	< 0.05, *
Monocyte CD11b	EF10175	1.12 (1.06-1.16)	< 0.01, **
	D39	1.13 (1.07-1.28)	< 0.05, *
Plasma MPO	EF10175	0.85 (0.58-1.13)	0.44, ns
	D39	0.81 (0.70-1.05)	0.22, ns
PNCs	EF10175	1.30 (1.19-1.62)	< 0.01, **
	D39	1.08 (0.99-1.22)	0.16, ns
PMCs	EF10175	1.13 (0.92-1.47)	0.32, ns
	D39	0.86 (0.74-1.03)	0.22, ns

[a] Values shown as median (IQR)

[b] Wilcoxon matched-pairs signed rank test

Supplementary Table 4. Relative fold increase by dispersed bacteria compared with biofilm bacteria in platelet-rich plasma

Marker	Strain	Dispersed/Biofilm [a]	*P* value [b]
CD62P	EF10175	3.96 (2.80-4.47)	< 0.01, **
	D39	1.22 (1.09-1.31)	0.06, ns
Plasma CD62P	EF10175	1.07 (0.96-1.12)	0.31, ns
	D39	0.99 (0.96-1.08)	> 0.99, ns
Plasma PF4	EF10175	0.83 (0.66-1.35)	0.84, ns
	D39	1.17 (0.74-1.39)	0.56, ns
CD63	EF10175	1.16 (1.03-1.19)	0.08, ns
	D39	1.07 (1.04-1.09)	0.13, ns
Mepacrine Release	EF10175	5.74 (2.96-13.00)	< 0.05, *
	D39	1.06 (1.01-13.02)	0.13, ns
Mepacrine Uptake	EF10175	0.23 (0.19-0.58)	< 0.05, *
	D39	0.89 (0.65-0.96)	0.13, ns

[a] Values shown as median (IQR)

[b] Wilcoxon matched-pairs signed rank test

1.2 Supplementary Figures

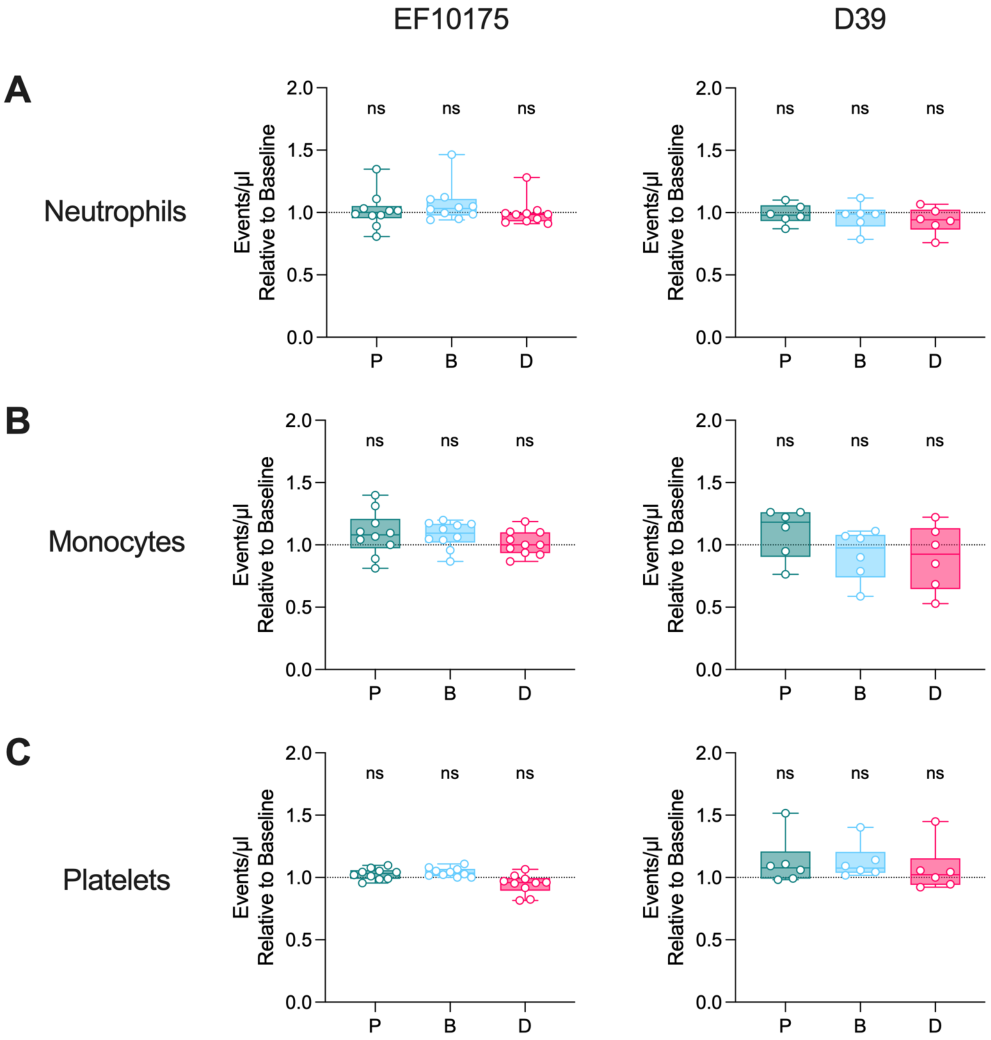

Supplementary Figure 1. Bacterial stimulation did not affect neutrophil, monocyte, and platelet counts. Citrated whole blood and diluted platelet-rich plasma (PRP) were stimulated with populations of planktonic (P), biofilm (B), or dispersed (D) pneumococci of strain EF10175 or strain D39 (1 x 10^7 CFU/ml) for 15 min at 37°C. The events/µl of **(A)** neutrophils, **(B)** monocytes, and **(C)** platelets were determined by flow cytometry. As a reference, the median events/µl for HEPES (baseline) samples was 201 (IQR 164-252) for neutrophils, 19 (IQR 16-23) for monocytes, and 1037 (IQR 911-1399) for platelets. EF10175, n = 10 independent experiments from 8 healthy donors; D39, n = 6 independent experiments from 5 healthy donors. Data are shown relative to baseline with the median ± interquartile range displayed. Statistical analysis was performed using Friedman test with Dunn's multiple comparisons test; ns = not significant.

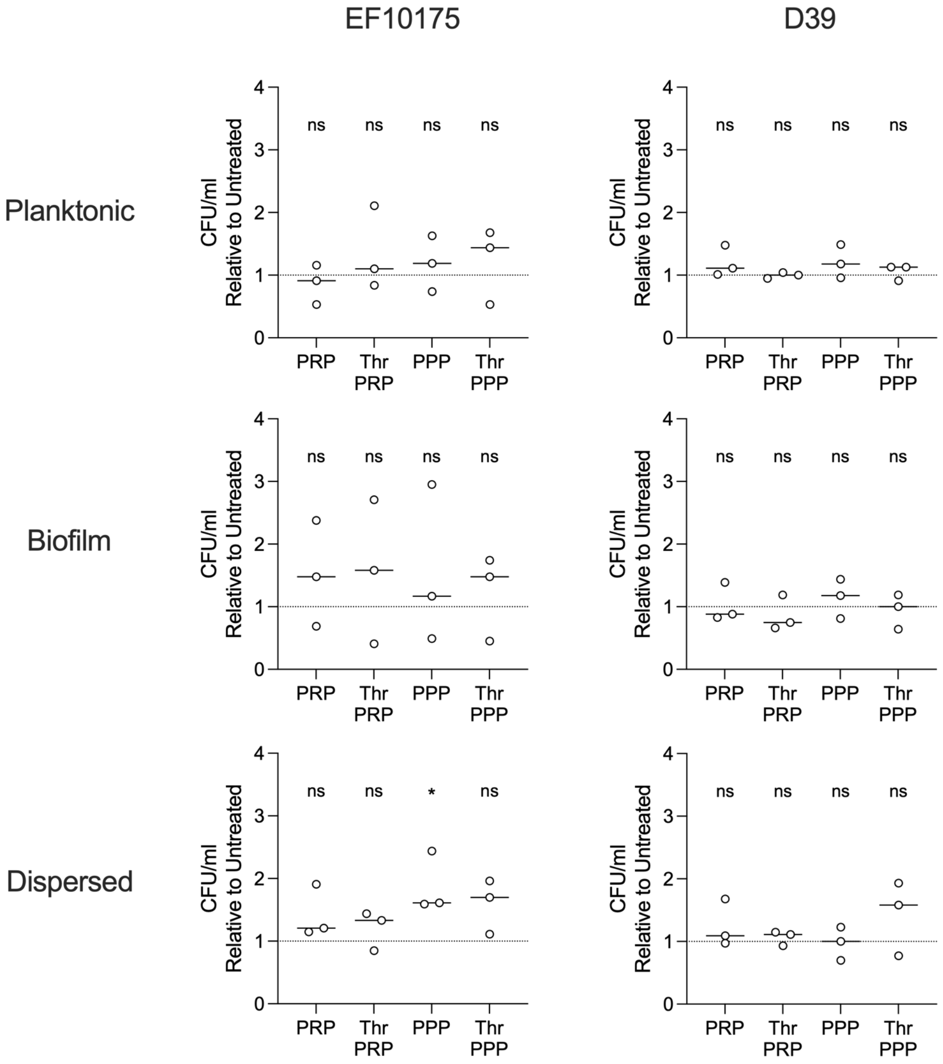

Supplementary Figure 2. Platelets and platelet releasate did not kill the pneumococcal populations. Populations of planktonic, biofilm, or dispersed pneumococci of strain EF10175 or strain D39 (1 x 10^7 CFU/ml) were incubated with diluted platelet-rich plasma (PRP), platelet-poor plasma (PPP), thrombin-stimulated PRP (Thr PRP) and respective PPP (Thr PPP), or HEPES (untreated) for 15 min at 37°C. Samples were plated for viable counts. n = 3 independent experiments from 3 healthy donors. Data are shown relative to untreated with the median ± interquartile range displayed. Statistical analysis was performed using Friedman test with Dunn's multiple comparisons test; ns = not significant, * $P < 0.05$.

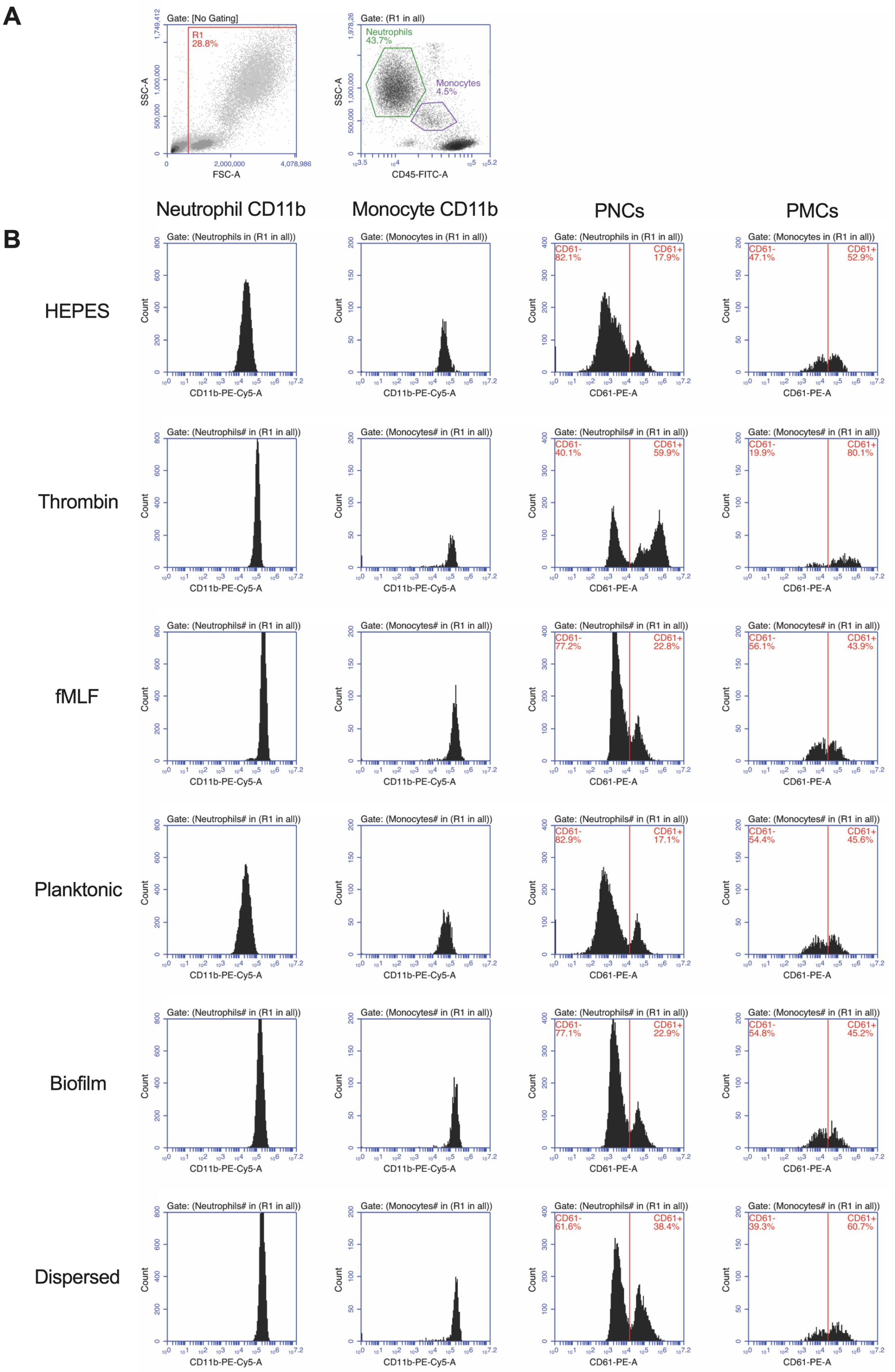
A
Gate: [No Gating]
R1
28.8%
SSC-A
FSC-A
Gate: (R1 in all)
Neutrophils
43.7%
Monocytes
4.5%
SSC-A
CD45-FITC-A
Neutrophil CD11b
Monocyte CD11b
PNCs
PMCs
B
HEPES
CD61-
82.1%
CD61+
17.9%
CD61-
47.1%
CD61+
52.9%
Thrombin
CD61-
40.1%
CD61+
59.9%
CD61-
19.9%
CD61+
80.1%
fMLF
CD61-
77.2%
CD61+
22.8%
CD61-
56.1%
CD61+
43.9%
Planktonic
CD61-
82.9%
CD61+
17.1%
CD61-
54.4%
CD61+
45.6%
Biofilm
CD61-
77.1%
CD61+
22.9%
CD61-
54.8%
CD61+
45.2%
Dispersed
CD61-
61.6%
CD61+
38.4%
CD61-
39.3%
CD61+
60.7%
Count
CD11b-PE-Cy5-A
CD61-PE-A

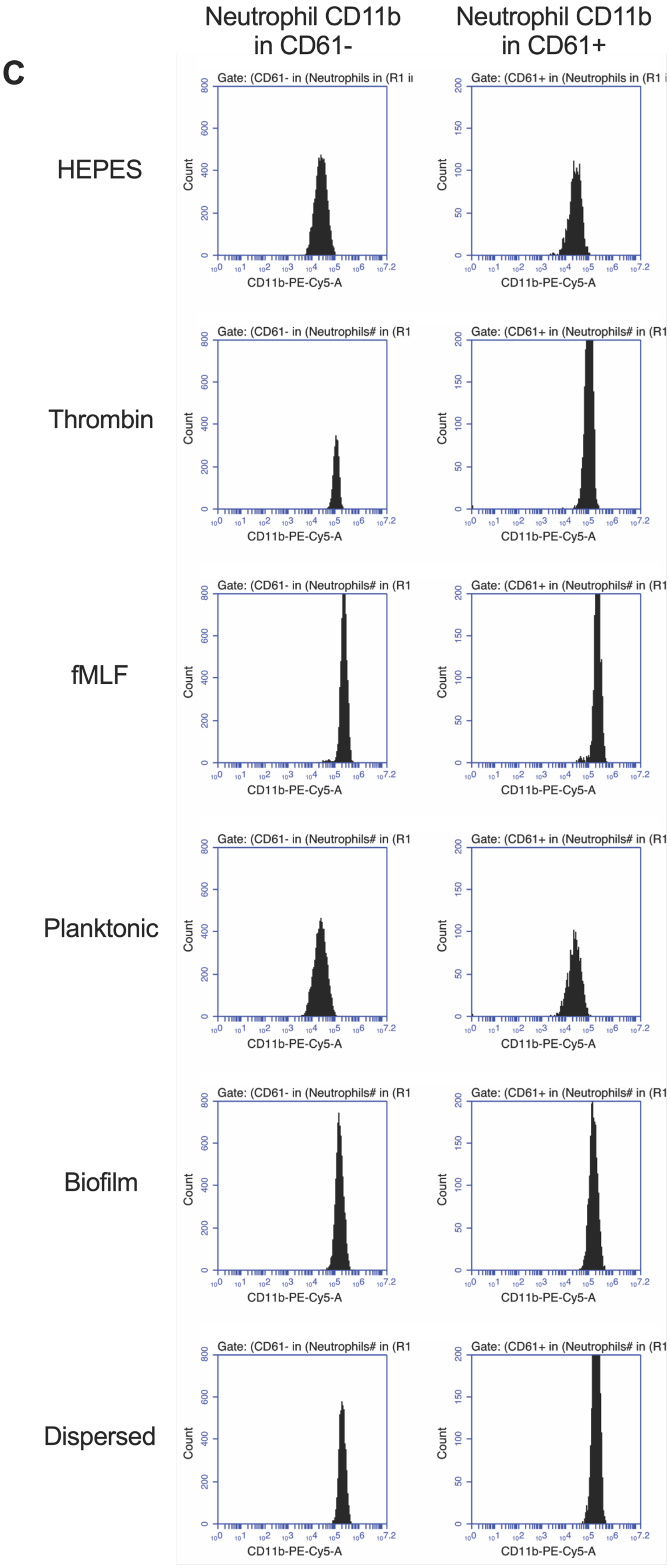
C
Neutrophil CD11b in CD61-
Neutrophil CD11b in CD61+
HEPES
Thrombin
fMLF
Planktonic
Biofilm
Dispersed
Gate: (CD61- in (Neutrophils in (R1 i
Gate: (CD61+ in (Neutrophils in (R1 i
Gate: (CD61- in (Neutrophils# in (R1
Gate: (CD61+ in (Neutrophils# in (R1
Count
CD11b-PE-Cy5-A

D

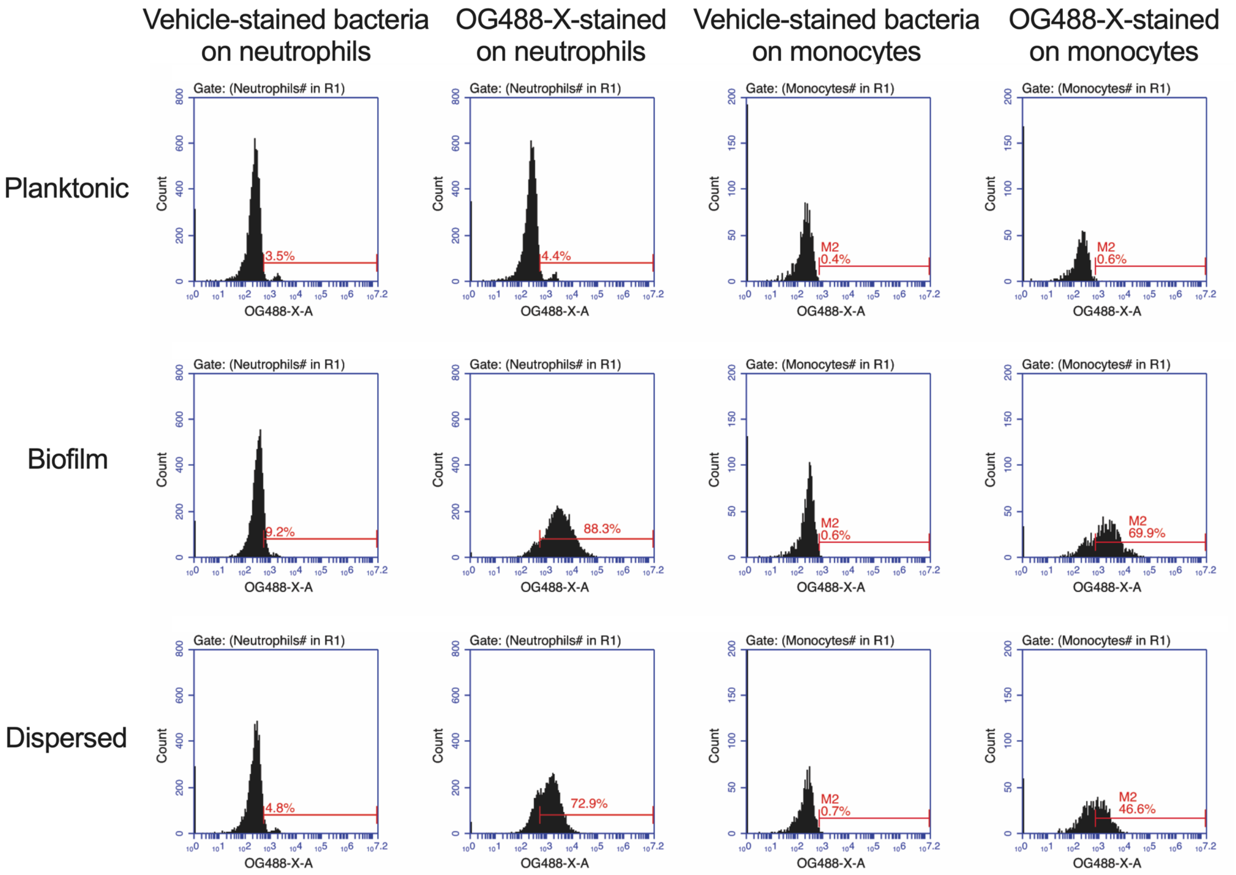

Vehicle-stained bacteria on neutrophils
OG488-X-stained on neutrophils
Vehicle-stained bacteria on monocytes
OG488-X-stained on monocytes
Planktonic
Biofilm
Dispersed
Gate: (Neutrophils# in R1)
Gate: (Monocytes# in R1)
Count
OG488-X-A
3.5%
4.4%
M2 0.4%
M2 0.6%
9.2%
88.3%
M2 0.6%
M2 69.9%
4.8%
72.9%
M2 0.7%
M2 46.6%

E

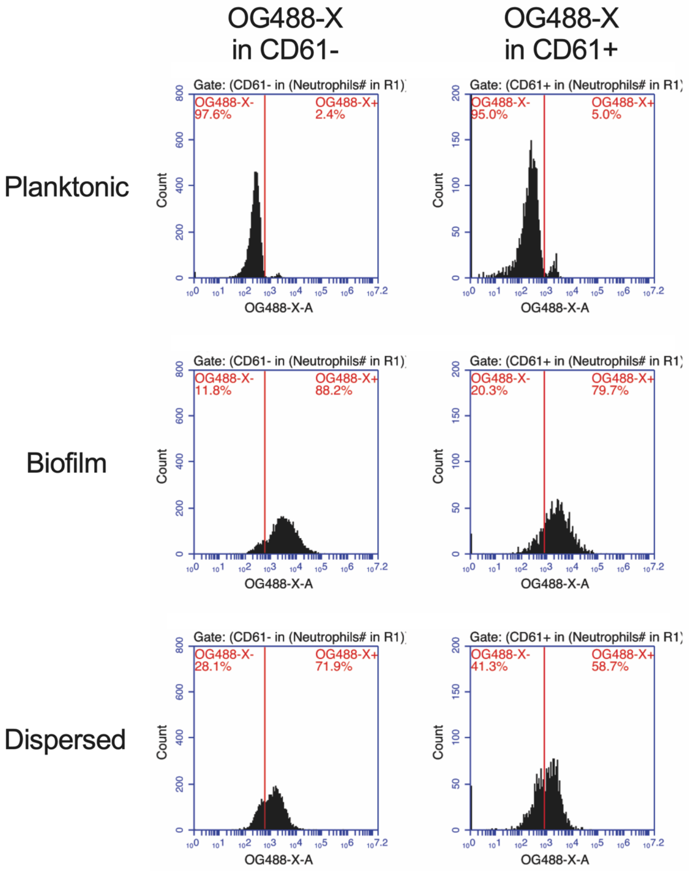

OG488-X in CD61-
OG488-X in CD61+
Planktonic
Biofilm
Dispersed
Gate: (CD61- in (Neutrophils# in R1)
Gate: (CD61+ in (Neutrophils# in R1)
Count
OG488-X-A
OG488-X- 97.6%
OG488-X+ 2.4%
OG488-X- 95.0%
OG488-X+ 5.0%
OG488-X- 11.8%
OG488-X+ 88.2%
OG488-X- 20.3%
OG488-X+ 79.7%
OG488-X- 28.1%
OG488-X+ 71.9%
OG488-X- 41.3%
OG488-X+ 58.7%

Supplementary Figure 3. Representative gating strategies and histograms for whole blood. **(A)** Leukocytes were gated using a linear scale FSC-A/SSC-A scatter plot with a FSC-H threshold of 150,000 to exclude red blood cell debris. Neutrophils and monocytes were gated based on a CD45-FITC-A/SSC-A (or CD45-APC/SSC-A) scatter plot. **(B)** The median fluorescence intensity (MFI) of neutrophil and monocyte activation (CD11b-PE-Cy5) and platelet-positive events (for PNCs and PMCs; CD61-PE) at baseline (HEPES) or with agonists (1 U/ml thrombin or 1 μM fMLF) or pneumococcal populations (1 x 10^7 CFU/ml) were determined based on histogram plots. **(C)** The MFI of neutrophil activation (CD11b-PE-Cy5) in the neutrophil CD61-negative and CD61-positive gates from **(B)** were determined based on histogram plots. **(D)** The percentage of neutrophils and monocytes stained with OG488-X or vehicle control were determined based on histogram plots. **(E)** The percentage of OG488-X (bacteria-positive events) in the neutrophil CD61-negative and CD61-positive gates from **(B)** were determined based on histogram plots.

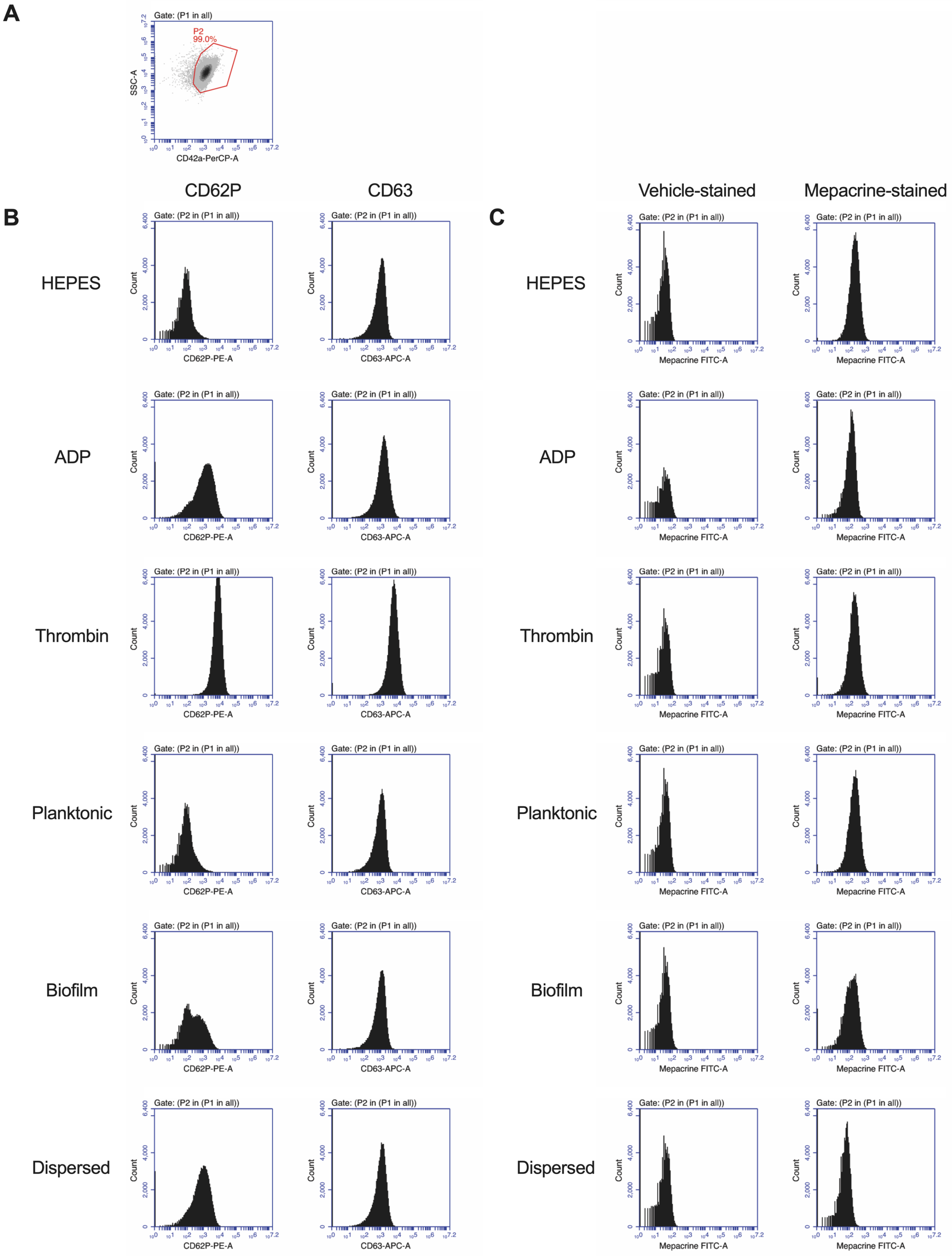
A
Gate: (P1 in all)
P2
99.0%
SSC-A
CD42a-PerCP-A
B
C
CD62P
CD63
Vehicle-stained
Mepacrine-stained
HEPES
ADP
Thrombin
Planktonic
Biofilm
Dispersed
Gate: (P2 in (P1 in all))
Count
CD62P-PE-A
CD63-APC-A
Mepacrine FITC-A

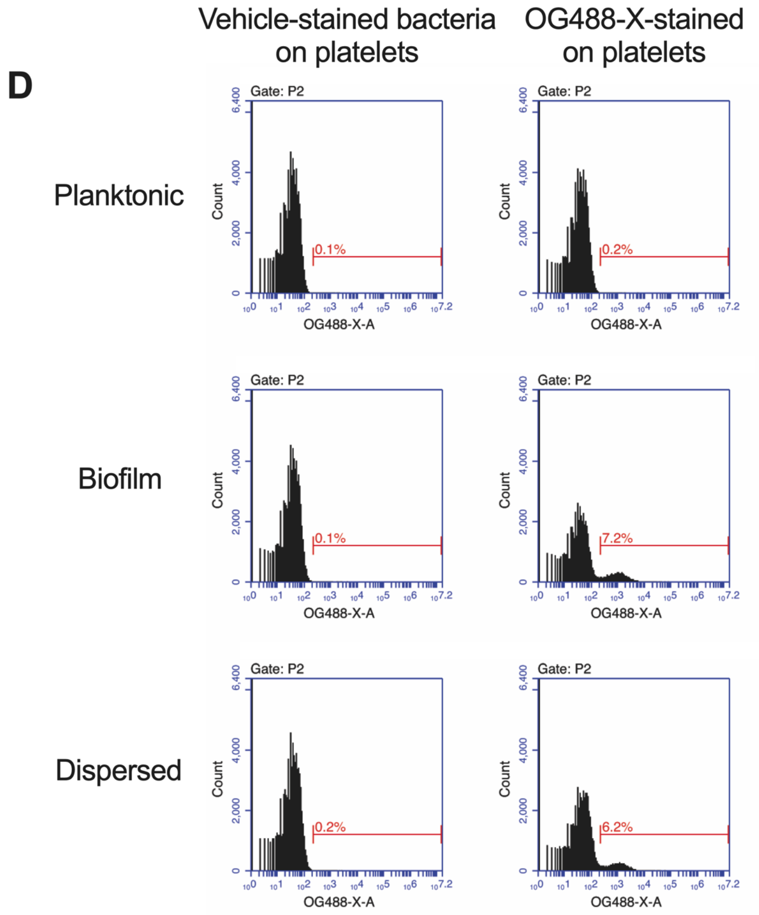

Supplementary Figure 4. Representative gating strategies and histograms for platelet-rich plasma. **(A)** Platelets were gated based on a CD42a-PerCP-A/SSC-A scatter plot. **(B)** The median fluorescence intensity (MFI) of activated platelets (CD62P-PE, CD63-APC) at baseline (HEPES) or with agonists (5 µM ADP or 1 U/ml thrombin) or pneumococcal populations (1 x 10^7 CFU/ml) were determined based on histogram plots. **(C)** The MFI of platelets stained with mepacrine or vehicle control were determined based on histogram plots. **(D)** The percentage of platelets stained with OG488-X or vehicle control were determined based on histogram plots.

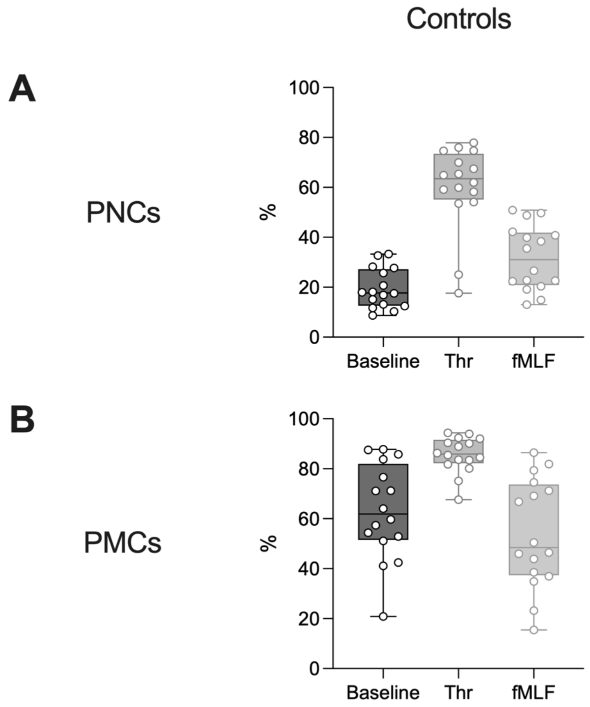

Supplementary Figure 5. % PNC and % PMC formation in whole blood. Citrated whole blood was stimulated with HEPES (to determine baseline levels), 1 U/ml thrombin, or 1 μM fMLF for 15 min at 37°C. The percentage of CD61 (platelet-positive events) on **(A)** neutrophils and **(B)** monocytes were determined by flow cytometry. Data are shown with the median ± interquartile range.

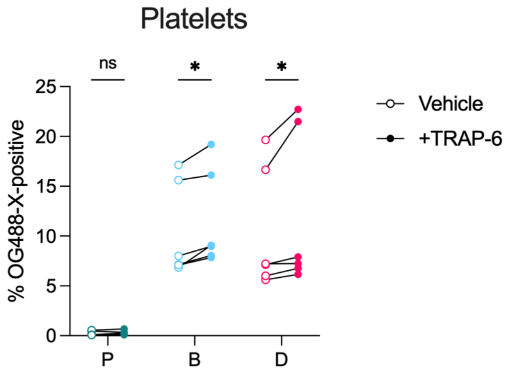

Supplementary Figure 6. Bacterial association with platelets after subsequent stimulation with TRAP-6. Diluted platelet-rich plasma was stimulated with non-stained or OG488-X-stained populations of planktonic (P), biofilm (B), or dispersed (D) pneumococci of strain EF10175 (1 x 10^7 CFU/ml) for 15 min at 37°C and incubated for an additional 5 min at 37°C in the presence of 20 µM TRAP-6 or vehicle control. The percentage of OG488-X-positive platelets were determined by flow cytometry. Bacteria association was calculated as the difference between the percentages of samples stimulated with OG488-X-stained bacteria and non-stained bacteria. n = 6 independent experiments from 6 healthy donors. Statistical analysis was performed using Wilcoxon matched-pairs signed rank test; ns = not significant, * $P < 0.05$.
